# A Google Earth Engine-enabled Python approach for the identification of anthropogenic palaeo-landscape features

**DOI:** 10.12688/openreseurope.13135.2

**Published:** 2021-09-03

**Authors:** Filippo Brandolini, Guillem Domingo-Ribas, Andrea Zerboni, Sam Turner

**Affiliations:** 1 McCord Centre for Landscape - School of History, Classics and Archaeology, Newcastle University, UK, Newcastle upon Tyne, NE4 5HP, UK; 2 Dipartimento di Scienze della Terra "Ardito Desio", Università degli Studi di Milano, Milano, I-20133, Italy

**Keywords:** Multispectral analysis, Sentinel-2, Spectral decomposition, Python, Riverscape, Fluvial and Alluvial Archaeology, Landscape Archaeology, Buried features

## Abstract

The necessity of sustainable development for landscapes has emerged as an important theme in recent decades. Current methods take a holistic approach to landscape heritage and promote an interdisciplinary dialogue to facilitate complementary landscape management strategies. With the socio-economic values of the “natural” and “cultural” landscape heritage increasingly recognised worldwide, remote sensing tools are being used more and more to facilitate the recording and management of landscape heritage. The advent of freeware cloud computing services has enabled significant improvements in landscape research allowing the rapid exploration and processing of satellite imagery such as the Landsat and Copernicus Sentinel datasets. This research represents one of the first applications of the Google Earth Engine (GEE)  Python application programming interface (API) in studies of historic landscapes. The complete free and open-source software (FOSS) cloud protocol proposed here consists of a Python code script developed in Google Colab, which could be adapted and replicated in different areas of the world. A multi-temporal approach has been adopted to investigate the potential of Sentinel-2 satellite imagery to detect buried hydrological and anthropogenic features along with spectral index and spectral decomposition analysis. The protocol's effectiveness in identifying palaeo-riverscape features has been tested in the Po Plain (N Italy).

## Introduction

### Toward a definition of “landscape heritage”

Landscapes emerge through complex interrelated natural and cultural processes and consequently encompass rich data pertaining to the long-term interactions between humans and their environments. Over recent millennia, human activities have become progressively more important in shaping geomorphic change ^1^ to the extent that some scientists argue Earth’s history has entered a new epoch, the Anthropocene ^2^. In this context, humans are active geomorphological agents, able to modify the physical landscape and shape anthropogenic landscape features ^3^. Multi-temporal analysis of landscape dynamics can help identify how human economic development, land use change and population growth have altered natural resources. Past landscape reconstruction enables a better understanding of human resilience to climatic and environmental changes in different periods and locations, and may illustrate examples of sustainable development in the past. At the same time, the analysis of historic land use permits the evaluation of human impact on natural environments ^4^. The importance of considering landscape’s “natural” and “cultural” heritage values together and promoting interdisciplinary approaches to develop conservation strategies has emerged increasingly strongly over the last decade ^5, 6^. This interdisciplinary perspective is epitomised in the Council of Europe’s European Landscape Convention which defines landscape as ‘an area, as perceived by people, whose character is the result of the action and interaction of natural and/or human factors’. This international treaty lays out pathways towards sustainable development in the landscape based on a balanced and harmonious relationship between social needs, economic activity and the environment. Identifying landscape heritage represents the first crucial phase in any conservation plan ^7– 9^. In this regard, modern GIS and remote sensing tools have become indispensable tools for landscape research which facilitate the mapping of territories over multiple spatial and temporal scales ^10^.

### GIS and remote sensing in landscape studies: the FOSS-cloud ‘revolution’

Geographic information systems (GIS) and remote sensing technologies are increasingly being recognized as effective tools for the documentation and management of valuable natural and cultural landscape features ^11, 12^. In particular, satellite remote sensing technologies have enabled significant improvements in landscape research and triggered the development of new tools in disciplines including Ecology ^13^, Geomorphology ^14, 15^ and Archaeology ^16– 18^.

However, GIS proprietary software licenses limit access to broader community growth and implementation, especially in developing nations. Conversely, FOSS (free and open source software) geospatial data and tools represent an invaluable alternative mitigating the need for software licensing and data acquisition, which is a critical barrier to broader participation ^10, 19, 20^. A further step toward more inclusive and borderless access to geospatial research is represented by freeware cloud computing services that enable users to process data and create outputs without significant investment in the hardware infrastructure ^21, 22^. The two main freeware cloud-based planetary-scale platforms available are the Google Earth Engine (GEE) and the Microsoft Planetary Computer.

The advent of GEE has enabled the rapid exploration and processing of more than 40 years of satellite imagery ^23^. GEE combines a multi-petabyte catalogue of geospatial datasets and provides a library of algorithms and a powerful application programming interface (API) ^24^. GEE eased the access to publicly available satellite imagery and earth observation tools in many branches of scientific research ^23, 25– 27^, revealing new opportunities especially for landscape heritage applications ^28^. Amongst others, GEE users can access Landsat (from 1972) and Sentinel (from 2014) datasets. The highest resolution available in GEE (up to 10 m/pixel) is offered by the Copernicus Sentinel-2 satellite constellation, which represents an invaluable free and open data source to support sustainable and cost-effective landscape monitoring ^29^. Sentinel-2 carries an innovative wide swath high-resolution multispectral imager (MSI) with 13 spectral bands providing information useful for a wide range of applications such as agricultural and forest monitoring ^30, 31^. Many studies have considered the potential of Sentinel-2 data in the cultural heritage domain at diverse scales of analysis, from single site up to landscape level ^32– 34^, and as a tool for scientific investigation and heritage management and preservation.

GEE can be employed in several main ways, including i) via the JavaScript API on the web-based IDE Earth Engine Code Editor, or ii) via Python API on local machines. A third option consists of using the Python API in Google Colaboratory (commonly referred to as “Colab”) ^35, 36^, a Python development environment that runs in the browser using Google Cloud. The Python API did not originally support any kind of visual output, but this limit has been quickly overcome with the development of new Python modules. Python has proven to be the most compatible and versatile programming language as it supports multi-platform application development. Finally, Python is continuously improved thanks to the implementation of new libraries and modules ^37^. Whilst the potential of Python in modelling landscape dynamics has been widely explored ^38, 39^, few publications have so far documented the use of the GEE Python API ^40^. In this paper we propose a complete FOSS-cloud approach to detect palaeo-landscape features through the GEE Python API in Colab.

### Why riverscapes?

The potential of using the GEE Python API in Colab has been tested in this paper on riverine landscapes for a number of reasons. Human activities have often relied on river systems, whether for agriculture, navigation or trade purposes. Fluvial/alluvial environments have been crucial since prehistory owing to the fertility of alluvial landforms and the availability of water supporting settlement, agriculture, mobility and trade ^41^. Archaeological investigations have confirmed that over the last 5000 years human activities have profoundly altered the spatial configuration and rate of fluvial processes, often inducing profound changes to river geomorphology ^42^. Riverine landscapes are excellent examples of landscapes which develop through complex relationships between human activities and environmental factors ^43– 45^. Moreover, the large scale of buried features such as river palaeochannels or ancient canals eases the identification of palaeo-riverscape features using remote sensing. In recent years, remote sensing and satellite imagery have been successfully applied to identify palaeo-geomorphological features (fluvial avulsion, fluvial channels, abandoned meanders, crevasse splays, backswamps) and anthropogenic structures (canals, irrigation systems, artificial levees) in many parts of the world ^46– 51^.

To assess the effectiveness of our FOSS-cloud protocol, northern Italy’s Po Plain was used as an ideal test case for the methodology. A huge amount of field- and remotely-sensed geomorphological data are available for the Po Plain and the whole region has been settled and exploited since the Neolithic period. The potential offered by Sentinel-2 imagery has recently been exploited here to map arable land ^52^. In this paper we attempt the first Python application of Sentinel-2 data for heritage research in a European riverscape and illustrate the possibility of detecting and interpreting buried anthropogenic landscape features originating in different periods.

### Test case area

The Po Plain (Northern Italy) results from the infilling of the depression between the Alps and the Apennines; it is the largest floodplain in Italy. The region forms a natural bridge between the Mediterranean and continental and eastern Europe, and is consequently a key area for understanding environmental and cultural connections between different contexts ^53^. People have been closely engaged with fluvial and alluvial dynamics since the region was first colonised and have actively shaped the geomorphology of the basin’s rivers since later prehistory.

### Geographic and geomorphological background

**Figure 1.**
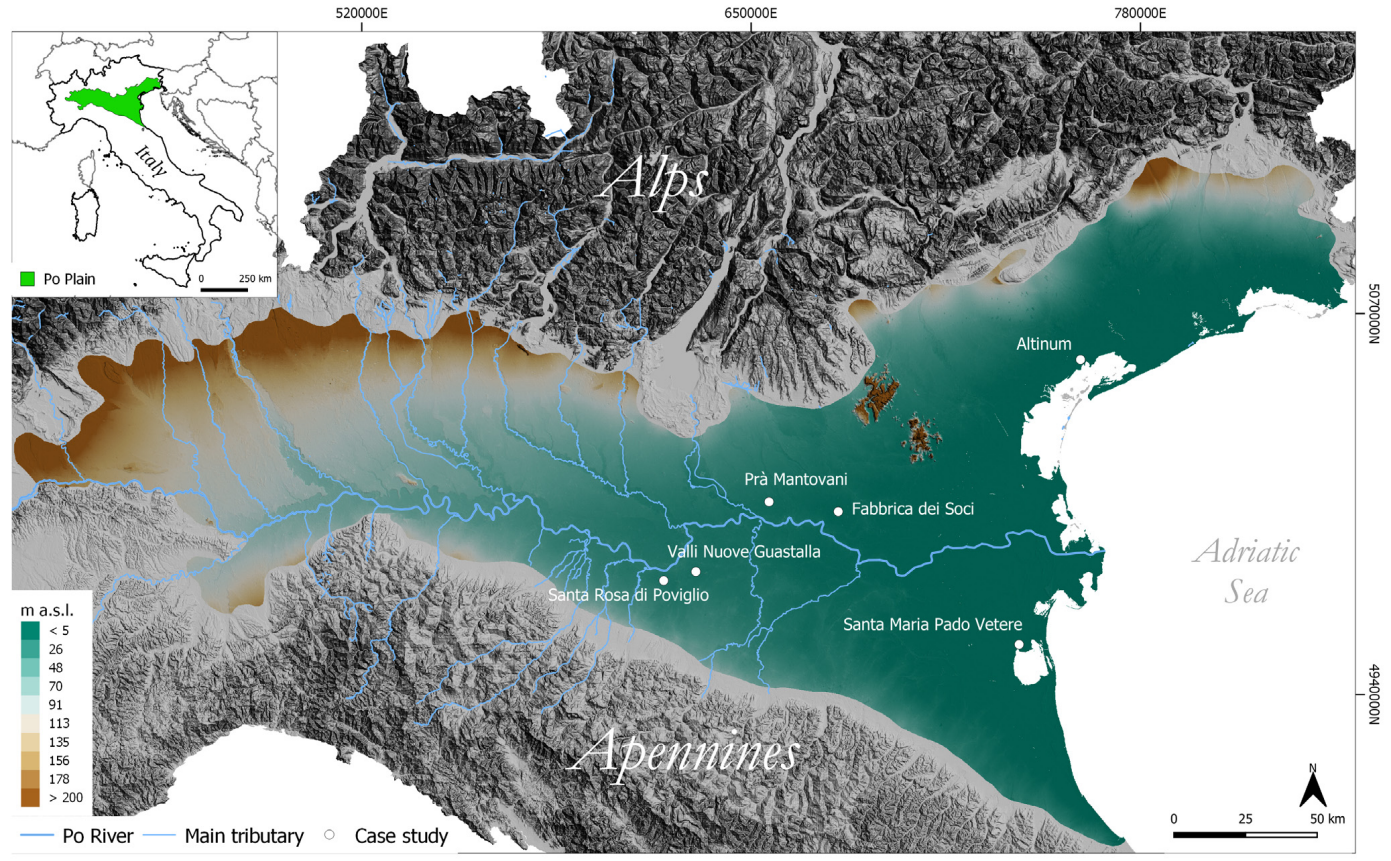
Schematic representation of the test case area.

The Po Plain and its eastward continuation – the Venetian-Friulan Plain – are situated in a transitional region between the Mediterranean and the European continental climate zones ( Figure 1). As reported in the Köppen classification, the Po Plain is characterised by a range from humid continental (Cfb) to humid subtropical (Cfa) climate ^54^. Intense rainfall (700–1200 mm per year) occurs throughout the year and the seasonal pattern of precipitation strongly influences the annual regime of the Po River ^55^. The highest rainfall is reached in spring and autumn while the lowest precipitation is usually registered in January and summer (June and July) ^56^. Peaks in the Po River discharge volumes are usually observed in late spring, likely due to melting snow from mid-altitude mountains ^57^.

The high levels of relative humidity are a consequence of the specific physiography of the plain, surrounded by the Alps and the Apennines, and the influence of the Adriatic Sea ^58, 59^ ( Figure 1). The geomorphological characteristics of the northern and southern sides of the plain differ profoundly ^60, 61^.

The area along the foothills of the Alps is characterized by the presence of Quaternary glacial amphitheatres ^62, 63^ in front of which fluvial fans slowly degrade southwards and eastwards. The fans are interpreted as a result of the mobilisation of glacial and fluvioglacial sediments by rivers which have formed an outwash plain over time ^64^. Different phases of alternating depositional and erosional events have resulted in the formation of terraced landforms along the outwash plains. The southern portion of this area consists of a succession of fluvial terraces shaped by the Po River and its tributaries and dating from the Upper Pleistocene to the Holocene ^65, 66^. Moving eastward, a large portion of the Po Plain and the Friulan-Venetian Plain were built by aggradation processes during the Last Glacial Maximum (LGM, ~22ka - 16ka years BCE) ^67, 68^. After that phase the Alpine tributaries of the Po River underwent a dramatic phase of incision that caused the formation of terraces and a downstream shift in deposition zones ^69^. On the opposing, southern side of the Po Plain, the Apennine watercourses developed an apron of fluvial mega-fans along the boundary of the floodplain. A well-preserved system of Late Pleistocene to Holocene alluvial fans extends northward between the Apennine foothills and the Holocene plain ^70, 71^. The distal part of alluvial fans presents a telescopic shape resulting from alternating aggradation/entrenchment phases tuned by Holocene climatic changes. Each aggradational cycle triggered an incision at the top of the pre-existing fan and the progradation of a new fan in a more distal position ^72^. Finally, during the Late Holocene, the aggradation of riverbeds resulted in channel diversions and frequent inundation of flood-prone areas ^73^. Additionally, in the eastward portion of the Po Plain and in the Venetian–Friulian area the Late Quaternary floodplain evolved in response to the climate-controlled development of alluvial systems and sea-level changes ^74– 76^.

### Environmental history and human settlements

Thanks to its complex settlement and land-management history, the Po Plain represents an ideal setting to assess the potentiality of our FOSS-cloud approach to detect riverscapes’ palaeo-features.

Since the Mid-Holocene (~5–3ka BCE), Neolithic communities settled at an increasing rate in the Po Plain owing to its suitability for agriculture ^77^. During the Bronze Age (~1700 – 1150 BCE), the Po Plain witnessed the emergence of proto-urban civilizations – the Terramare culture – that altered the natural fluvial landscape, introducing the earliest systems for hydraulic management of the fluvial network and extensive woodland clearance ^78– 80^. Deforestation and farming development heightened during the Iron Age (~1100–700 BCE) – the Etruscan period – when agricultural activities became the major land use and farmers the key agents in modifying the landscape ^81, 82^. Between the 2nd-1st century BCE, the Po Plain was modified significantly following Roman colonisation, with the introduction of the centuriation system for agricultural management which entailed the creation of a regular grid of roads, ditches and fields. In this phase, at least 60% of the surface of the Po Plain was deforested and converted into farmland ^83^. From the 5th century CE, a lack of maintenance of irrigation networks which may have been linked to political disruption associated with the end of the Roman Empire ^84^, combined with surface instability triggered by a cool climate phase ^85^, meant that large portions of the Po Plain changed into wetlands ^70^ *.* This progressive waterlogging process endured until the beginning of the 10th century CE with significant implications for settlement and farming practices ^86^. Between the 10th and 14th centuries CE – corresponding to the Medieval Warm Period ^85^– land reclamation intensified owing to an increased demand for arable land alongside general population growth in Europe ^87^. At the beginning of the 12th century CE wetland reclamation, the construction of levees and canalisation increased and a series of canals were constructed in the Po Plain for irrigation and navigation ^88, 89^. In the Renaissance, extensive land and water management activities advanced the process of land reclamation in many coastal and interior wetlands ^73, 90^. During the Little Ice Age (~1500–1850 CE ca.) deforestation accelerated and reached its peak in the late 1700s, while the construction of embankments was completed during the 19th century CE ^85^. Flood defences and drainage systems were further reinforced during the 20th century to reduce the risk of inundation ^91^. Human water/land management and natural resource exploitation (e.g. deforestation and quarrying) have been so widespread over the centuries that only a tiny portion of this riverscape can be considered completely ‘natural’ today ^83^.

## Material and methods

The first application of GIS and remote sensing techniques to record the past landscape settings of the Po Plain dates back to the end of the nineties ^92^. Today, significant improvements in FOSS software and the increased availability of open-source satellite datasets enable the development of more efficient remote sensing approaches.

The mosaic of cultivated ﬁelds on the Po floodplain is subject to frequent changes which can make uniform visual analysis difficult ^50^; this heterogeneity can also complicate the detection of past riverscape features, as the factors that influence it (crop types, seasonal rainfall, soil moisture) vary in areas with different environmental conditions. For example, variations in the capacity to retain soil moisture are a major factor precluding or enhancing the detection of ancient hydrological features ^34, 93^. Multi-temporal datasets have the capacity to include diverse land-use/land-cover (LULC) scenarios enabling identification of features that may not be visible on individual images during a particular period of the year ^50^.

### Sentinel-2 dataset

The Sentinel-2 (S2) satellite constellation was developed by the European Space Agency (ESA) in the framework of the European Commission Copernicus Programme. The twin satellites (A and B) of the S2 programme have a 5-day temporal resolution and their multispectral sensors acquire data in 13 separate bands with a spatial resolution up to 10 m ( Table 1). In this paper we utilize the GEE dataset S2 MSI (MultiSpectral Instrument), Level-1C orthorectified top-of-atmosphere (TOA) reflectance (dataset availability: June 2015 - present) filtered with the cloud masking quality assurance band QA60.

**Table 1.** S2 Satellites bands properties. ( https://sentinel.esa.int/web/sentinel/technical-guides/sentinel-2-msi/msi-instrument).

Name	Pixel Size	Wavelength	Description
B1	60 meters	443.9nm (S2A) / 442.3nm (S2B)	Aerosols
B2	10 metres	496.6nm (S2A) / 492.1nm (S2B)	Blue
B3	10 metres	560nm (S2A) / 559nm (S2B)	Green
B4	10 metres	664.5nm (S2A) / 665nm (S2B)	Red
B5	20 metres	703.9nm (S2A) / 703.8nm (S2B)	Red Edge 1
B6	20 metres	740.2nm (S2A) / 739.1nm (S2B)	Red Edge 2
B7	20 metres	782.5nm (S2A) / 779.7nm (S2B)	Red Edge 3
B8	10 metres	835.1nm (S2A) / 833nm (S2B)	NIR
B8A	20 metres	864.8nm (S2A) / 864nm (S2B)	Red Edge 4
B9	60 metres	945nm (S2A) / 943.2nm (S2B)	Water Vapor
B10	60 metres	1373.5nm (S2A) / 1376.9nm (S2B)	Cirrus
B11	20 metres	1613.7nm (S2A) / 1610.4nm (S2B)	SWIR 1
B12	20 metres	2202.4nm (S2A) / 2185.7nm (S2B)	SWIR 2

Buried natural palaeochannels and human structures result in crop marks and soil marks on the surface because they retain a different amount of moisture compared to the surrounding soil ^94^. The identification of crop/soil marks from aerial imagery has informed the identification of buried archaeological sites since the 1920s ^95– 97^. Satellite multispectral images can be more effective in this respect than traditional aerial photography and researchers have identified key bands for the detection of palaeo-landscape features: visible (0.4 – 0.7 µm), near infrared (NIR) (0.7 – 1.4 µm), and short-wave infrared (SWIR) (1.4 – 3 µm) ^32, 98, 99^.

Even with the high resolution of modern satellite sensors ^100^, the detection of crop marks is often affected by several issues, the most important being the phenological stage of the crops ^101, 102^. The heterogeneity of the Po Plain farmland and high annual precipitation rates further complicate the recognition of crop marks in the area. Meanwhile soil marks can appear on bare soil as colour changes, easily identifiable after ploughing: differences in soil colour in ploughed farmland highlight traces of past features whether positive (e.g. damper, wetter material from a palaeochannel or former ditch) or negative (e.g. buried natural or artificial levees) ^16^. In the case of the Po Plain previous studies ^103– 105^ suggested that buried features are likely to be more visible in soil marks after ploughing. Moreover, as highlighted in similar methodological studies, archaeological features tend to be more often visible on bare soil than in cultivated fields in S2 satellite imagery ^34^. 

Nevertheless, variations in local weather conditions or agriculture processes mean buried features appear in different ways in images acquired at different times. This problem applies to satellite (or aerial) imagery and can lead to negative impacts on the detection of crop or soil marks.

To help overcome this issue and to optimize the visibility of palaeo-features, this study adopted a multi-temporal approach ^50, 102^ by calculating the mean values of bands in the most promising periods for the identification of crop/soil marks between the years 2015 – 2020.

**Figure 2.**
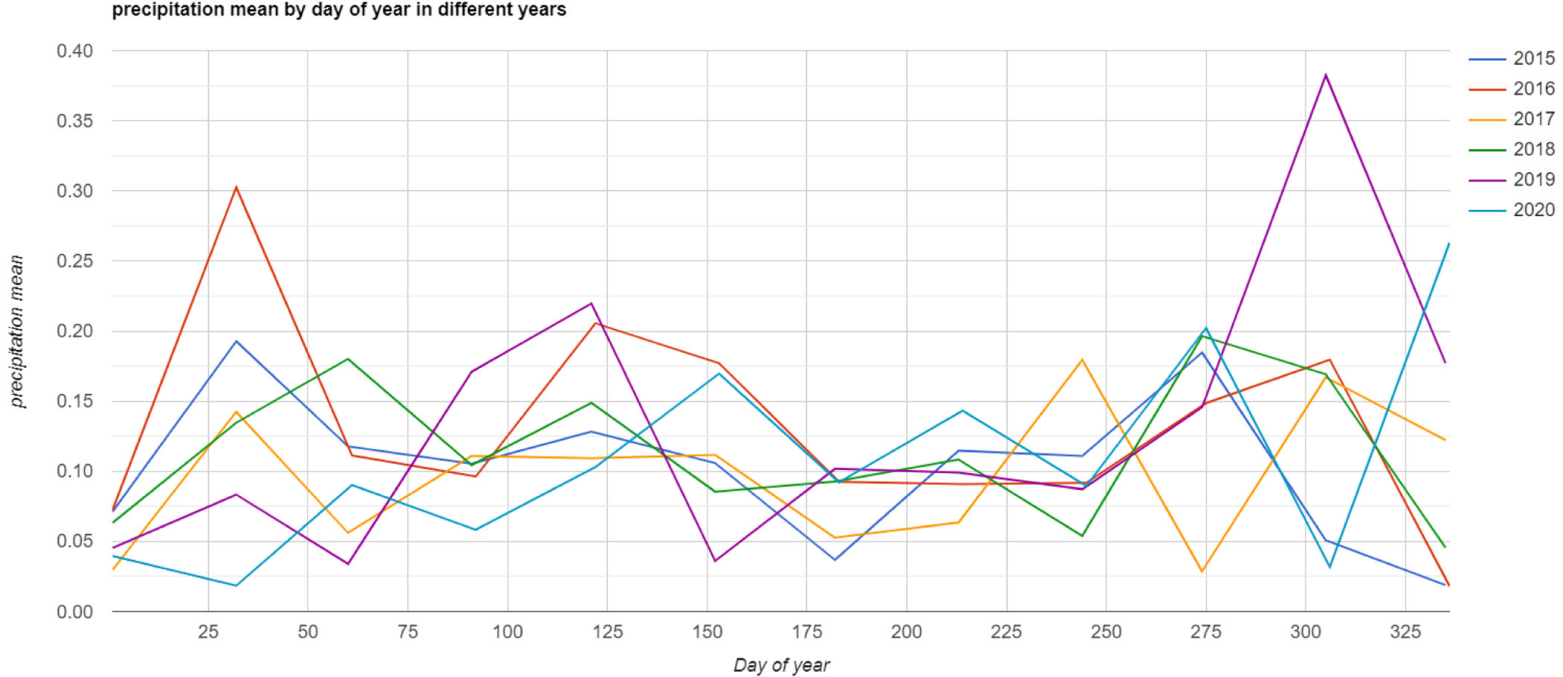
Annual mean precipitation rate between 2015 and 2020 in Po Plain. Source: Monthly Global Precipitation Measurement (GPM) v6. Plot generated in Google Earth Engine.

In the test case area the choice of timespan was driven by two specific environmental factors. The first is related to the increase in intensity and frequency of drought episodes in the Po Plain in the last decade ^56^: the longest recorded period of drought lasted from October 2016 to November 2017 ( Figure 2). As noted above, changes in soil moisture retention tends to facilitate the detection of crop/soil marks especially in severe drought periods.

**Figure 3.**
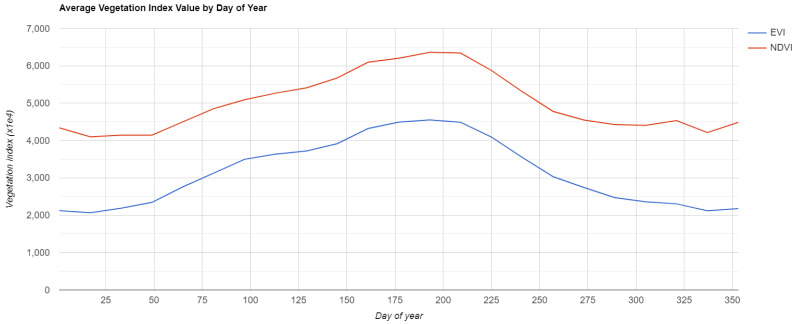
Average Vegetation Index Value by Day of Year between 2015 and 2020 in Po Plain using Normalized Difference Vegetation Index (NDVI) and Enhanced Vegetation Index (EVI). Source: MOD13A1.006 Terra Vegetation Indices 16-Day Global 500m. Plot generated in Google Earth Engine.

Secondly, autumn, winter and early spring are periods of relatively uniform land cover in the Po Plain ^104^: ploughing takes place across large areas of arable land, rice paddy fields have not yet been inundated and other winter crops have not yet reached their maximum growth ( Figure 3). Focusing on the low-vegetation period of the year the general uniformity in the land cover helps to mitigate the problems of field mosaicking, easing the detection of buried features.

**Figure 4.**
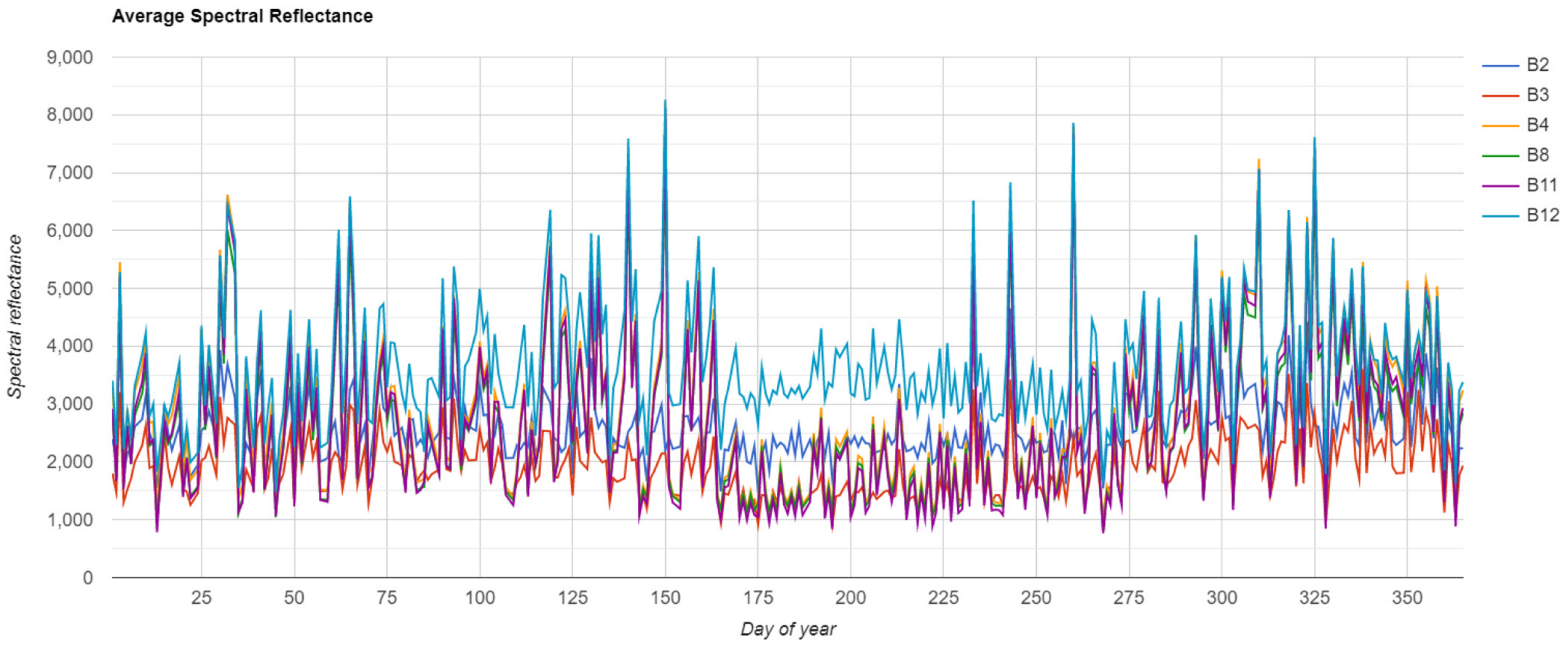
S2 Average Spectral Reflectance by Day of Year between 2015 and 2020. Plot generated in Google Earth Engine.

Furthermore, looking at the average annual performance of S2 bands, 150 - 270 days of the year (DoY) show a general lower average bands reflectance than other seasons: this is likely due to the fact that vegetation strongly absorbs radiation and the resulting reflectance is generally low ^106^ ( Figure 4).

The annual average vegetation cover and spectral reflectance helped in choosing the multi-temporal timespan that corresponds to two low vegetated periods (30 – 120 and 270 – 360 DoY) of each year from 2015–2020. The resulting image collections are merged and reduced in a single image. In other words, this workflow generates a single composite image for the entire analysis period containing six S2 bands (B2, B3, B4, B8, B11 and B12, see Table 1).

The choice of timespan is the only part of the protocol that needs to be customised by users according to the peculiar environmental conditions of each study area. In our test case area no preliminary bands sensitivity test ^33^ has been performed. This was beyond the scope of the current paper, whose the main goal is to evaluate the potential and limits of this cloud protocol as an alternative remote sensing FOSS-tool for mapping buried landscape features. Pre-existing literature about the occurrence of buried natural and anthropogenic features in the Po Plain provided a valuable set of benchmarks for evaluating the effectiveness of the method.

**Figure 5.**
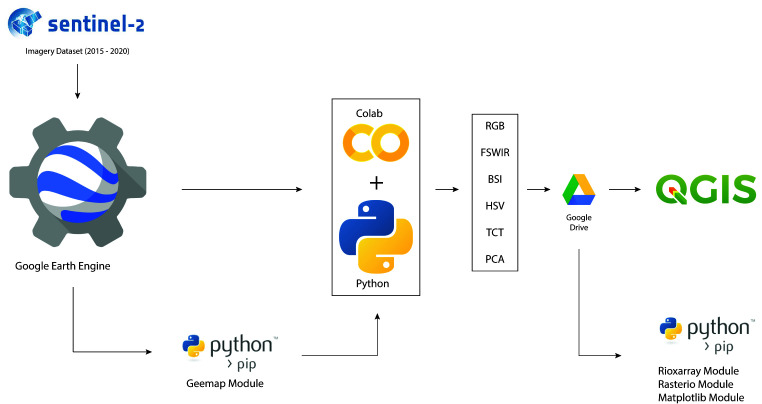
Free and open-source software (FOSS) methodological approach adopted in this research. RGB: Red-Green-Blue colour composite; FSWIR: False colour composite; BSI: Bare Soil Index; HSV: Hue, Saturation and Value; TCT: Tasselled Cap Transformation; PCA: Principal Component Analysis.

The S2 satellite data were accessed through the Python module geemap ^107^ in Colab ^35^, a serverless Jupyter notebook computational environment for interactive development. The native GEE Python API has relatively limited functionality for visualizing results but the geemap Python module was created specifically to fill this gap. The Python code developed enables the analysis of the S2 filtered image collection through spectral index (SI) and spectral decomposition (SD) techniques. Each image was exported in Geo.TIFF format in QGIS where the min/max values were adjusted with the cumulative count cut tool. Finally, the figures presented in this paper were generated in the QGIS layout editor. The Python module rasterio ^108^ was used to create individual plots for each band of the raster. Additionally, the Python packages rioxarray and matplotlib ^109^ were employed, respectively, to access each raster band and create customisable histograms of their values. ( Figure 5).

### Spectral indices

SIs for remote sensing purposes consist of mathematical combinations of different bands to enhance particular environmental characteristics. Their use is common in different fields of research, for example in monitoring variations in snow and glacier cover or in disaster prevention and management ^110^.

In this study, multi-temporal red-green-blue (RGB) colour composites were used to generate two different compositions: RGB (bands 4-3-2), and false short wave infrared colour (FSWIR, bands 12-8-4). RGB provides a true-colour visualization, very similar to the human colour perception, while false-colour images enable the identification of areas with different reflectance response to enhance the visibility of anomalies.

Spectral indices that combine NIR and red channels generally increase the visibility of crop- and soil-marks. Vegetation indices (VIs) have been widely tested to detect buried structure and fluvial palaeochannels ^34, 50, 99, 101, 111^. In particular, Agapiou *et al*. ^98^ reformulated the NDVI (normalized difference vegetation index) to elaborate a specific VI for the identification of archaeological remains: the normalized archaeological index (NAI). Focusing on the low-vegetation period of the year, this study adopted spectral indices that could potentially enhance the detection of soil marks including the bare soil index (BSI). The BSI combines blue (B2), red (B11), NIR (B8), and SWIR 1 (B4) spectral bands to capture soil variations ^112^ according to the formula: (( *red*+ *SWIR* 1) – ( *NIR*+ *blue*)) / (( *red*+ *SWIR* 1) + ( *NIR*+ *blue*)).

The SWIR and the red bands are employed to quantify the soil mineral composition, while the blue and the near infrared spectral bands enhance the vegetation. In general, the SWIR spectral range is strongly sensitive to soil moisture content enabling the detection of moisture variations in space and time ^16^; recent research suggests the SWIR2 band may be valuable for calculating BSI because it seems more sensitive in terms of classification accuracy ^113^. For this reason, the SWIR2 band was used in this study to calculate both FSWIR and BSI indices.

### Spectral decomposition

Three different spectral decomposition (SD) techniques were used in this study: hue, saturation and value (HSV), tasselled cap transformation (TCT) and principal component analysis (PCA). HSV, TCT and PCA have been successfully employed to detect both archaeological structure and past fluvial features in different environmental contexts ^34, 50, 114^. Here these three SD approaches were tested to detect past riverscape features in continental environmental conditions.

***Hue, saturation and value (HSV)***. HSV (hue, saturation, value, also known as HSB or hue, saturation, brightness) is an alternative representation of the RGB colour space. In HSV SD, Hue (H) defines pure colour in terms of red, green and blue, Saturation (S) specifies the purity of a colour relative to gray, and value (V) refers to the brightness of the colour. HSV performs a rotation from the RGB axis and it is characterized by the three relevant properties: 1- nonlinearity, 2- reversibility and 3 - independence of each component from the others ^16^. In our Colab Python script code, we calculate HSV through the GEE method . rgbToHsv().

***Tasselled cap transformation (TCT)***. The TCT, known also as Kauth-Thomas technique ^115^, was developed for enhancing spectral information content of satellite data. The TCT consists in a transformation of the original images into a new data set obtained by linear combinations of the original bands. This SD technique is performed on a pixel basis to better represent the underlying structure of the image according to the formula:

TC=(WTc)(DN)+B

where *WTc* stands for weighted transforming coefficient (i.e. speciﬁc transformation coefﬁcients statistically derived from images and empirical observations), *DN* for digital number and *B* for bias. The transformation *WTc* depends on the sensor considered, because different sensors have different numbers of bands which, in turn, have different spectral responses ^16^. There are three composite variables of TCT bands which are routinely adopted: *brightness* (TCTb, measure of bare soil), *greenness* (TCTg, measure of vegetation), *wetness* or *yellowness* (TCTw, measure of soil and canopy moisture) ^116^. To calculate the TCT bands for S2, the WTcs recently defined by Shi and Xu ^117^ were adopted for their better performance than previous proposed coefficient indexes ^118, 119^. Finally, in Colab, we computed the TCT components with the ee.Array type utilising the Sentinel-2 TCT Coefficients for the 6-Band Image (blue, green, red, NIR, SWIR1, SWIR 2) ( Table 2).

**Table 2.** WTcs for S2 defined by 100.

TCT bands	S2 WTcs
TCTb	0.3510 BLUE + 0.3813 GREEN + 0.3437 RED + 0.7196 NIR + 0.2396 SWIR 1 + 0.1949 SWIR 2
TCTg	-0.3599 BLUE -0.3533 GREEN -0.4734 RED + 0.6633 NIR - 0.0087 SWIR1 -0.2856 SWIR2
TCTw	0.2578 BLUE + 0.2305 GREEN + 0.0883 RED + 0.1071 NIR -0.7611 SWIR1 -0.5308 SWIR2

***Principal component analysis (PCA)***. The PCA transform (also known as the Karhunen-Loeve transform) consists of a linear transformation which decorrelates multivariate data by rotating the axes of the original feature space and outputs uncorrelated data ^120^. PCA reduced the dimensionality of the data, providing a new series of less correlated bands, limiting the loss of information and enhancing the features of interest ^16, 34^. In the Python script code the PCA is calculated by diagonalizing the input band correlation matrix through Eigen-analysis ( eigen()).

## Results

To assess the potential of the FOSS - cloud procedure discussed in this paper, the Python script code was tested at different locations in the Po Plain with well-known archaeological sites. The key points selected to test the script code consist of well-documented areas where anthropogenic activities have altered the pristine alluvial and fluvial geomorphological settings since protohistory. The case studies (from west to east) are: *Terramara Santa Rosa di Poviglio* (RE), *Valli Nuove di Guastalla* (RE), *Pra’ Mantovani* (MN), *Fabbrica dei Soci* (VR), *Santa Maria in Pado Vetere* (FE) and *Altinum* (VE) ( Figure 1).

### Santa Rosa di Poviglio

**Figure 6.**
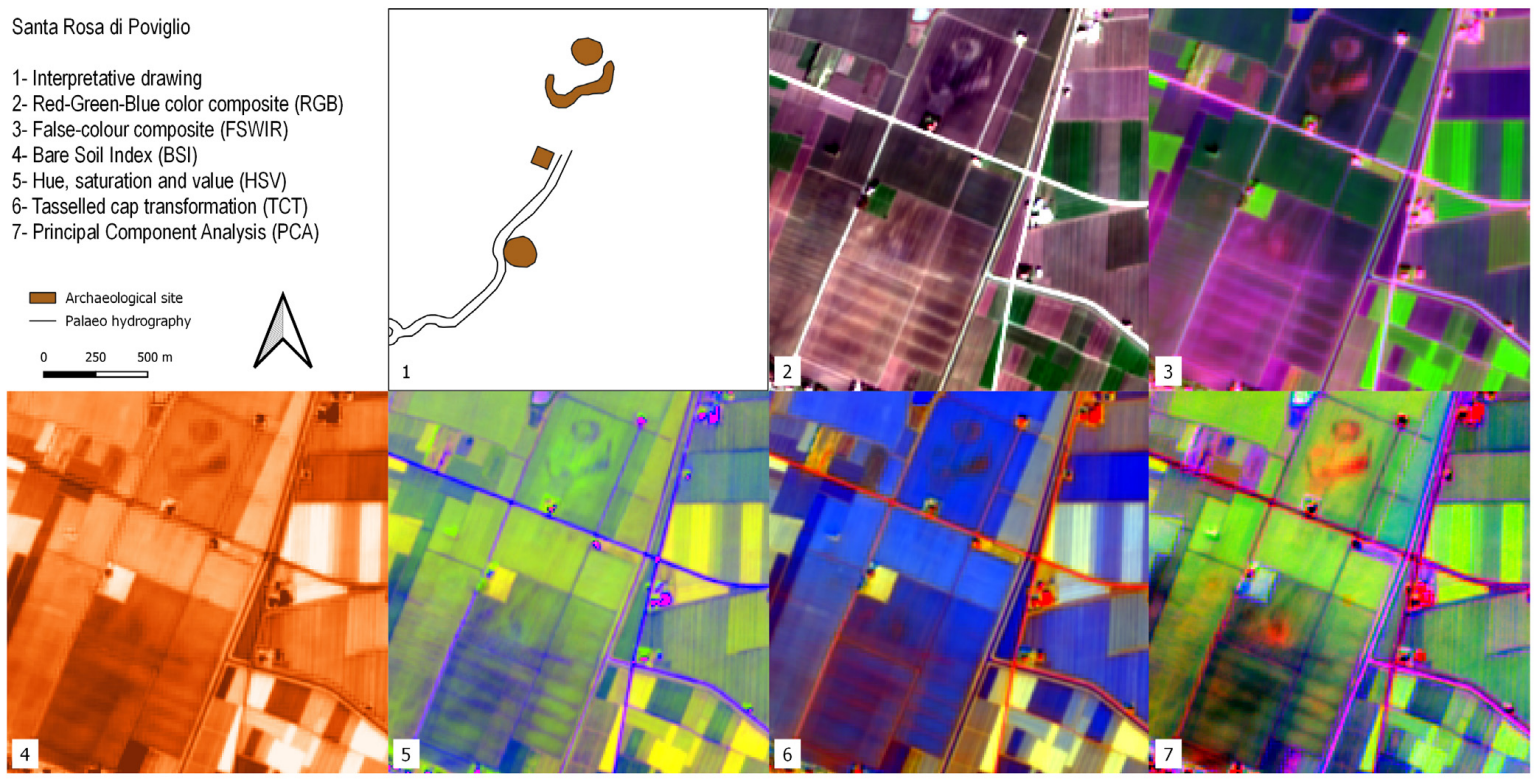
Outputs of the Terramara Santa Rosa di Poviglio site.

The site of Terramara Santa Rosa di Poviglio is a key settlement associated with the Bronze Age Terramare Culture (TC) ^121^. The village and its surroundings were delineated through an artificial modification of a pre-existing crevasse splay lobe. The settlement consists of two moated villages delimited by earth ramparts connected to an adjoining river channel through a canal network ^122, 123^. The earth ramparts are easily visible in all the SI and SD analysis performed as shown in Figure 6. The soil marks corresponding to the two moated villages are particularly evident in the FSWIR and BSI compositions while RGB, HSV and PCA images highlight the presence of a palaeochannel that flows southwards from the TC site. A square-shape feature lies near the southern limit of the Bronze Age village and corresponds to a Roman structure related to the centuriation of the surrounding landscape ^124^.

### Valli Nuove Guastalla

**Figure 7.**
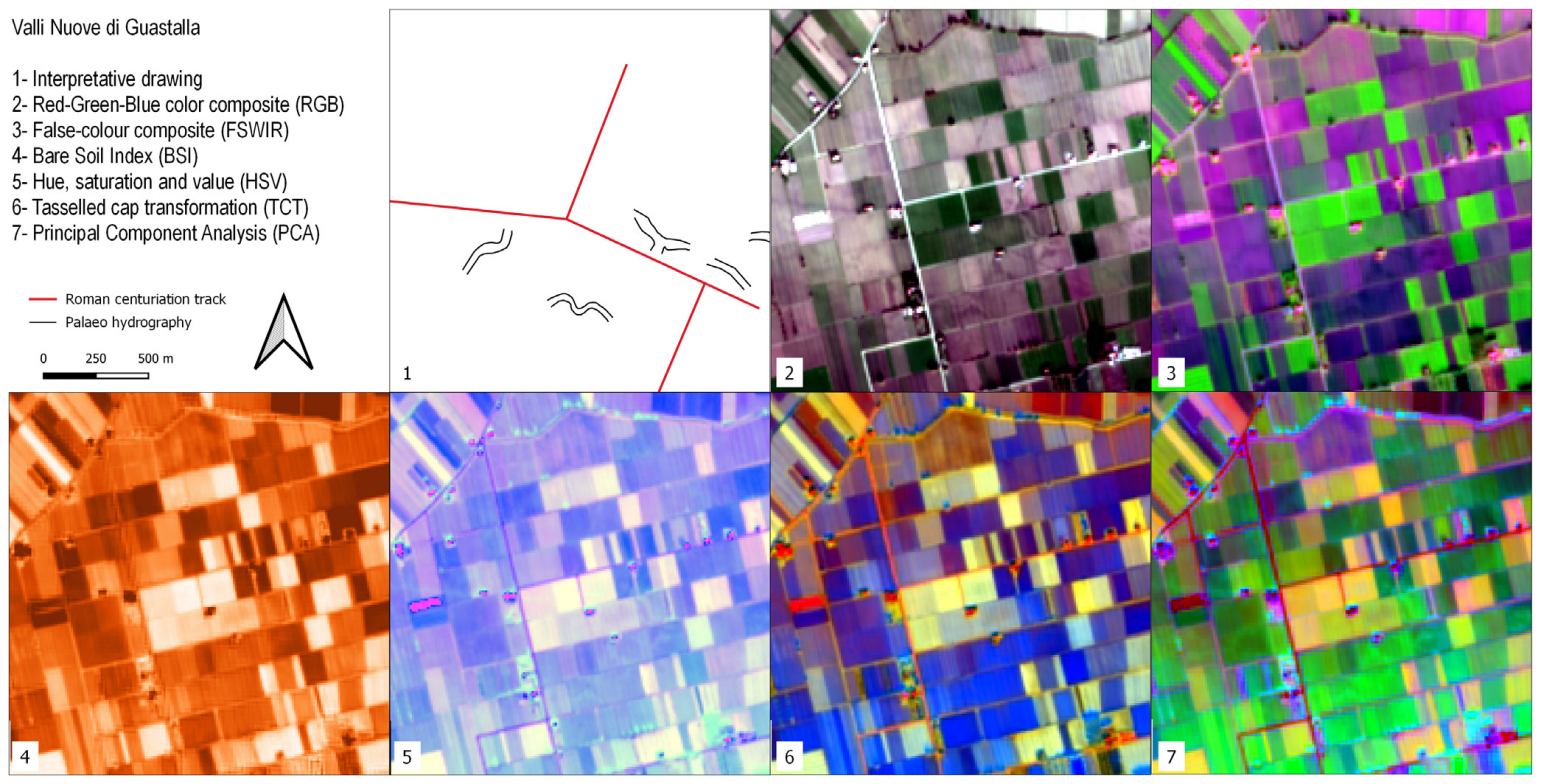
Outputs of the Valli Nuove di Guastalla site.

This site lies in the Central Po Plain, not far from the Terramara Santa Rosa di Poviglio site, in a portion of the floodplain known as “backswamp”. This geomorphological terms refers to the lowest area of floodplains, poorly drained, where finer sediments accumulate after flooding events ^42^. As noted above, the period which witnessed the collapse of the Roman Empire was also associated with climatic instability and progressive waterlogging of the Po Plain. The Roman farmland of the backswamps was inundated and became a palustrine environment ^73^. Valli Nuove Guastalla is a good location to investigate the impact of the processes which occurred between the Roman and the Medieval eras even though the cultivated mosaic of fields precludes clear visibility of crop/soil marks here ( Figure 7). In the RGB image calculated from the S2 seasonal mean values three buried orthogonal axes are barely visible, remnants of the drainage system created through Roman centuriation. These palaeofeatures are slightly visible also in the FSWIR and PCA images although hardly recognisable in the others: this condition is due mainly to the S2 image resolution as treated in detail in the Discussion. Buried canals and palaeochannels are highlighted in the FSWIR, HSV and PCA images: these features are most likely the results of flood management during Medieval land reclamation activities in the area ^73^.

### Pra’ Mantovani

**Figure 8.**
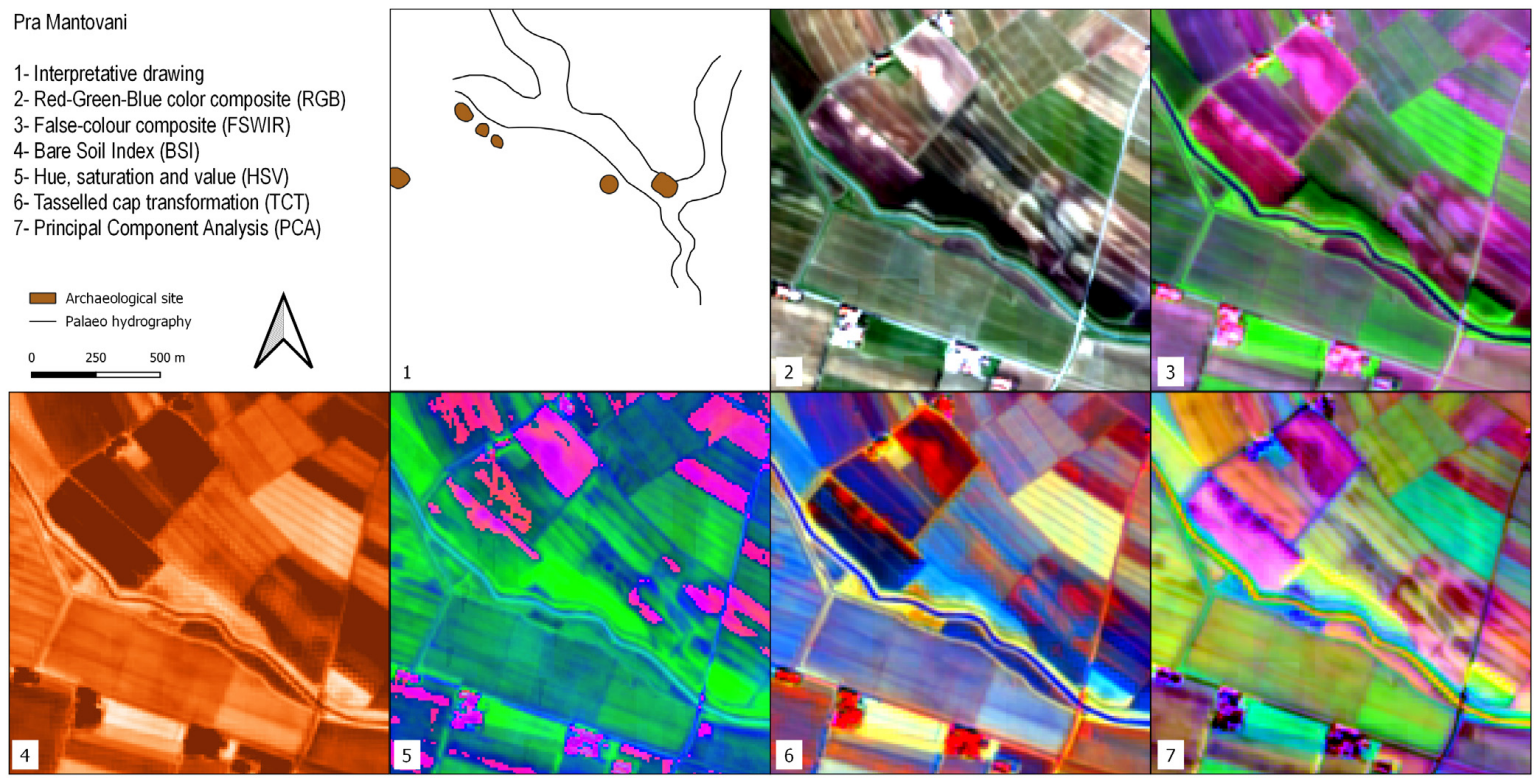
Outputs of the Pra’ Mantovani site.

The environmental context of the Pra’ Mantovani sites is similar to Valli Nuove di Guastalla. Here, recent archaeological surveys ^125, 126^ have registered the presence of Medieval settlements and buried Roman ditches. In all the SI/SD of Figure 8, an Early Medieval motte is clearly visible almost in the middle of the area. In the surroundings of this archaeological feature, a series of palaeochannels can be recognised. Positive crop and soils marks in the RGB and PCA images highlight irregular rounded features that have been interpreted as buried archaeological structures ^125, 126^.

### Fabbrica dei Soci

**Figure 9.**
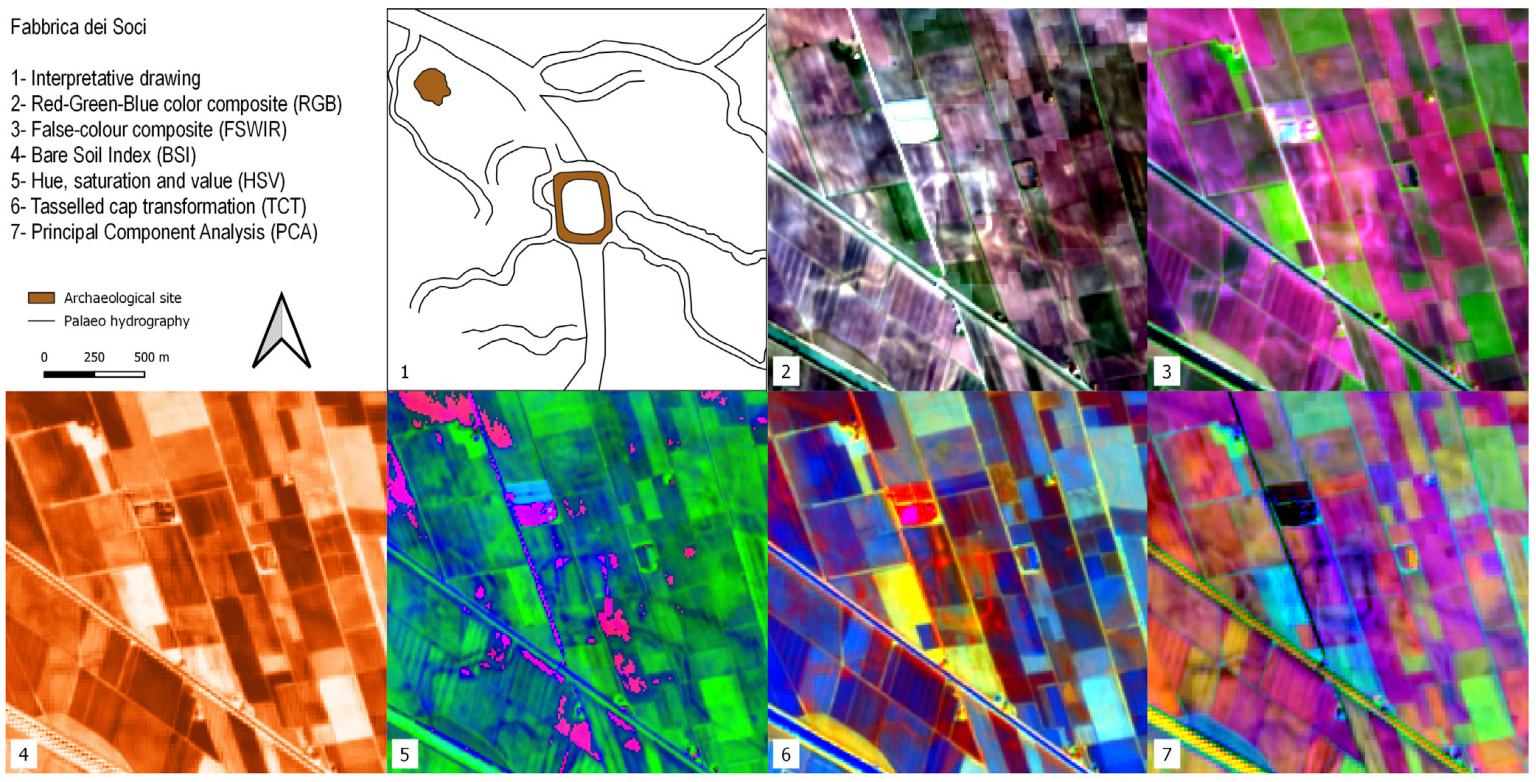
Outputs of the Fabbrica dei Soci site.

This site is one of the most important TC settlements in the Po Plain. In all the SI and SD images the general pattern of the site and the area nearby is clearly detectable ( Figure 9). The Terramara Fabbrica dei Soci presents a regular square-shaped village centred in a complex hydraulic system that distributed the water diverted from a river palaeo-channel in the surrounding fields for irrigation ^103^. The water management documented at this site can be considered as paradigmatic for the whole TC ^104, 127, 128^. Moats, canals and palaeochannels are especially recognisable in the RGB, FSWIR and PCA images. In the HSV image, the shape of the buried palaeochannels is particularly legible, while in the TCT the square-shaped settlement stands out clearly.

### Santa Maria in Pado Vetere

**Figure 10.**
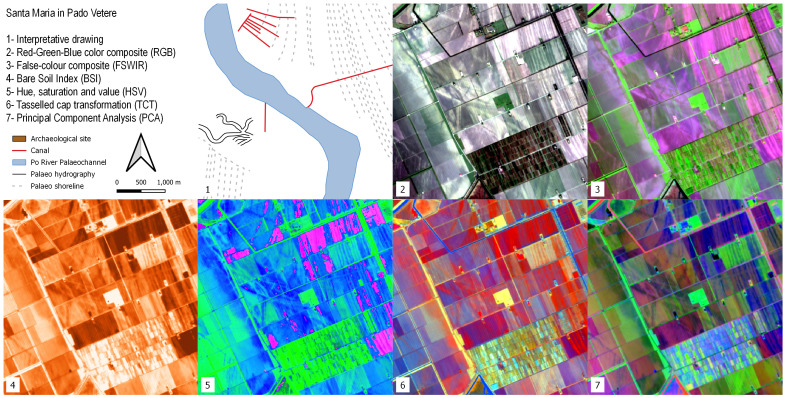
Outputs of the Santa Maria in Pado Vetere site.

Santa Maria in Pado Vetere consists of an Early Medieval church located in the area of the former palustrine environment known as *Valli di Comacchio* (FE). These backswamps were completely reclaimed during the 20th century CE ^129, 130^. The land reclamation works unearthed several archaeological sites, in particular the Etruscan harbour of Spina ^131, 132^, Roman villas and the early Medieval church of Santa Maria ^133– 135^. The place name “ *in Pado Vetere*” derives from the latin “ *Padus Vetus*” and indicates the presence of a Po River palaeochannel. This palaeo-riverscape feature is clearly visible in all the SI/SD images ( Figure 10) crossing the area from NW to SE. That course of the Po River flowed close to the Santa Maria church. Buried artificial canals are connected to the *Padus Vetus* and were probably used for navigation and irrigation purposes. The archaeological area of Spina and the Santa Maria church cemetery are hardly recognisable due to the resolution of the S2 imagery (see Discussion). In all the images, buried Holocene coastlines are easily detectable ^135, 136^. In the southern sector of the area the highly fragmented pattern of the farmland here precludes the visibility of the Po River palaeochannel and all other buried features: a similar situation was observed in the Valli Nuove di Guastalla.

### Altinum

**Figure 11.**
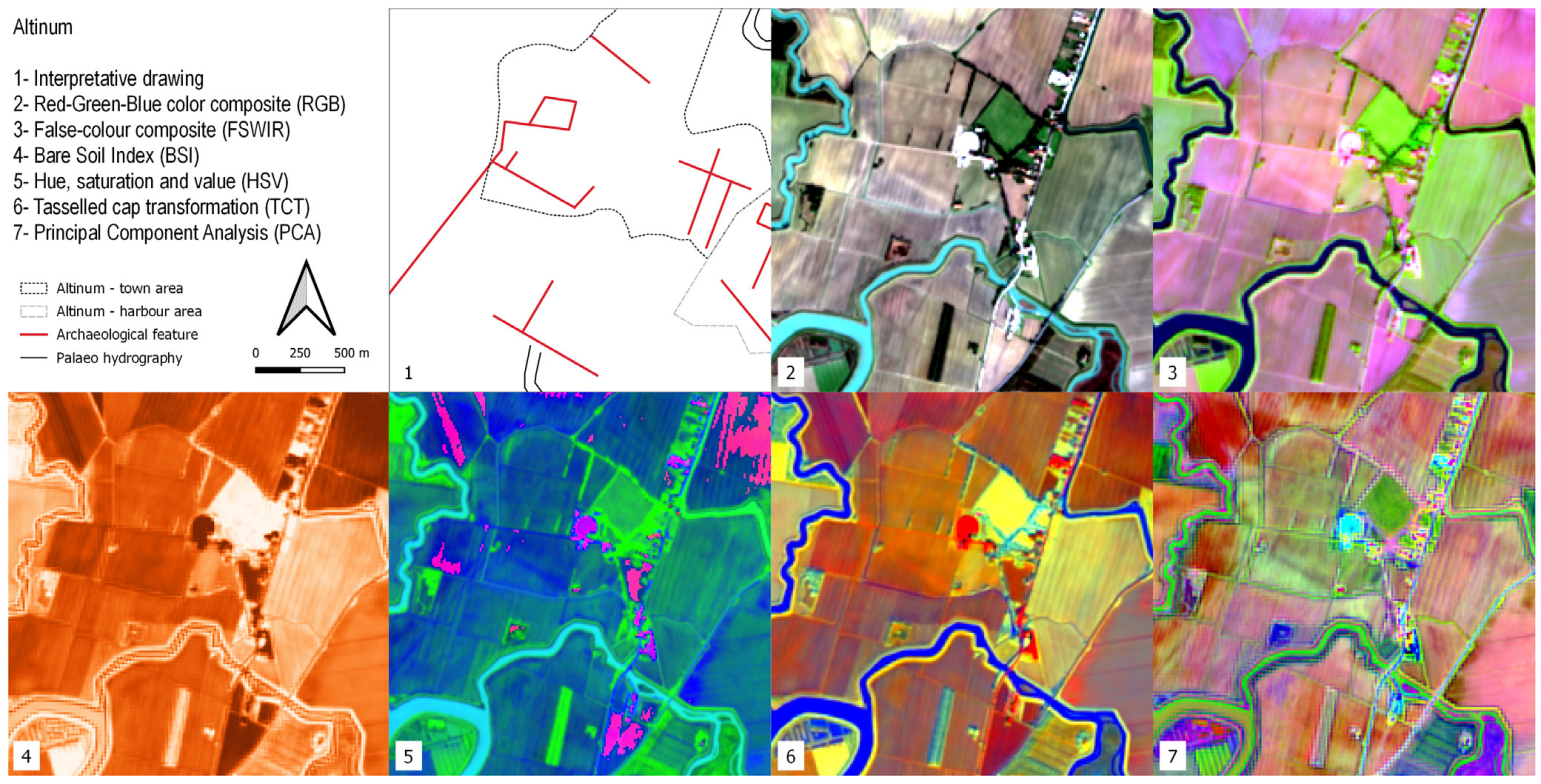
Outputs of the Altinum site.

Altinum was a Roman harbour on the inner margin of the Lagoon of Venice founded in the 1st century BCE. Its inhabitants colonized the northern lagoon islands in the 5th century CE and created the earliest settlement at Venice. This site was particularly suited for testing the Python script code because the features detected could be compared with the results of a study that reconstructed the urban topography and palaeoenvironmental setting of Altinum using high - resolution near-infrared (NIR) aerial photographs ^137^. Traces of buried hydrological features are visible in the area near the Roman city. Canals and roads are the only elements of the Altinum urban topography that can be detected with our FOSS-cloud protocol: as mentioned above, this limit is related to the resolution of the S2 bands. ( Figure 11).

## Discussion

The outputs generated for the test locations of the Po Plain show some of the potentialities and limits of the GEE Python API in Colab as an alternative remote sensing tool to identify buried natural and anthropogenic palaeo-riverscape features.

As noted previously, to identify the best period of visibility it is crucial to take into consideration crop rotation and meteorological conditions in the region of interest. In our test area the choice was particularly strategic because the autumn and winter/early spring seasons are characterised by a low vegetation and relatively uniform land cover ( Figure 3 and Figure 4). Moreover, the detection of crop/soil marks is strongly related to the soil moisture retention of buried features. In this regard, the S2 image collection selected includes severe drought events (e.g. years 2016 and 2017) alternated with higher precipitation rate periods (e.g. year 2018) ( Figure 2); this alternation of high and low rainfall intensity seems to have positively affected the calculation of the mean values of multitemporal bands for the identification of crop/soil marks. As we expected, the period selected to perform the multitemporal analysis proved fruitful in terms of detection of crop and (especially) soil marks. As known from the literature ^103– 105^, buried features (both natural and archaeological) appear to be more visible on bare soil than in cultivated fields, especially in highly mosaicised farmland. In the test area this eventuality was observed especially in the cases of Valli Nuove di Guastalla ( Figure 7) and Santa Maria Pado Vetere ( Figure 10).

**Figure 12.**
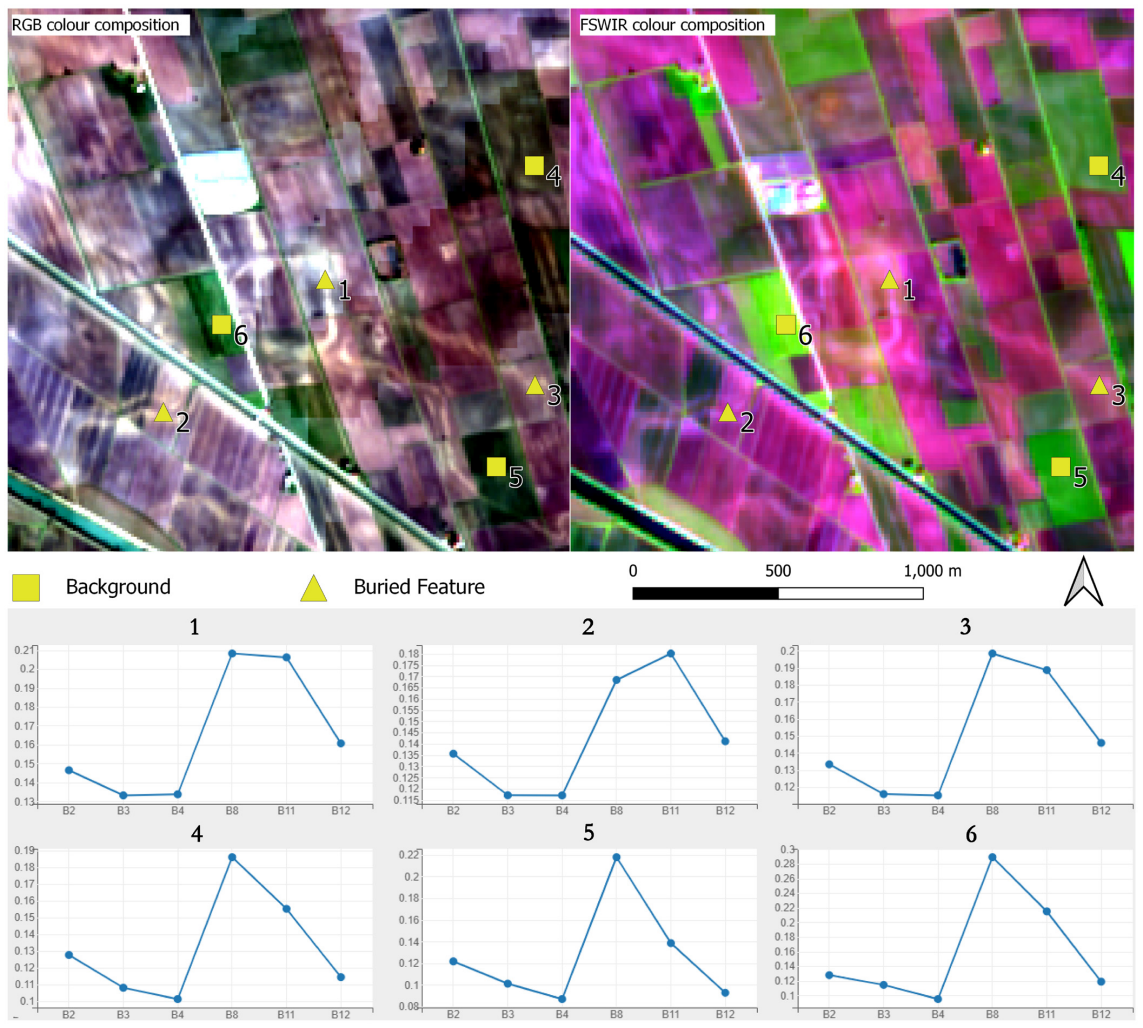
Fabbrica dei Soci site: spectral signature of individual Sentinel-2 bands sample in correspondence of paleo features and in the neighbouring background.

The Inspector and Plotting tools of the geemap Python module enable the extraction of spectral signature value of pixels interactively. Basically, users can sample spectral values directly on the displayed outputs. For example, Figure 12 shows the application of these tools in the Fabbrica dei Soci site ( Figure 9). The analysis of the spectral signatures between the buried palaeo-features and neighbouring area shows significant differences in the B11 (SWIR 1) and B12 (SWIR 2) values. As the literature shows ^16, 113^, the SWIR spectral range is strongly sensitive to soil moisture content easing the detection of moisture variations: in our test area SWIR bands seem particularly effective in the detection of buried palaeo-features.

**Figure 13.**
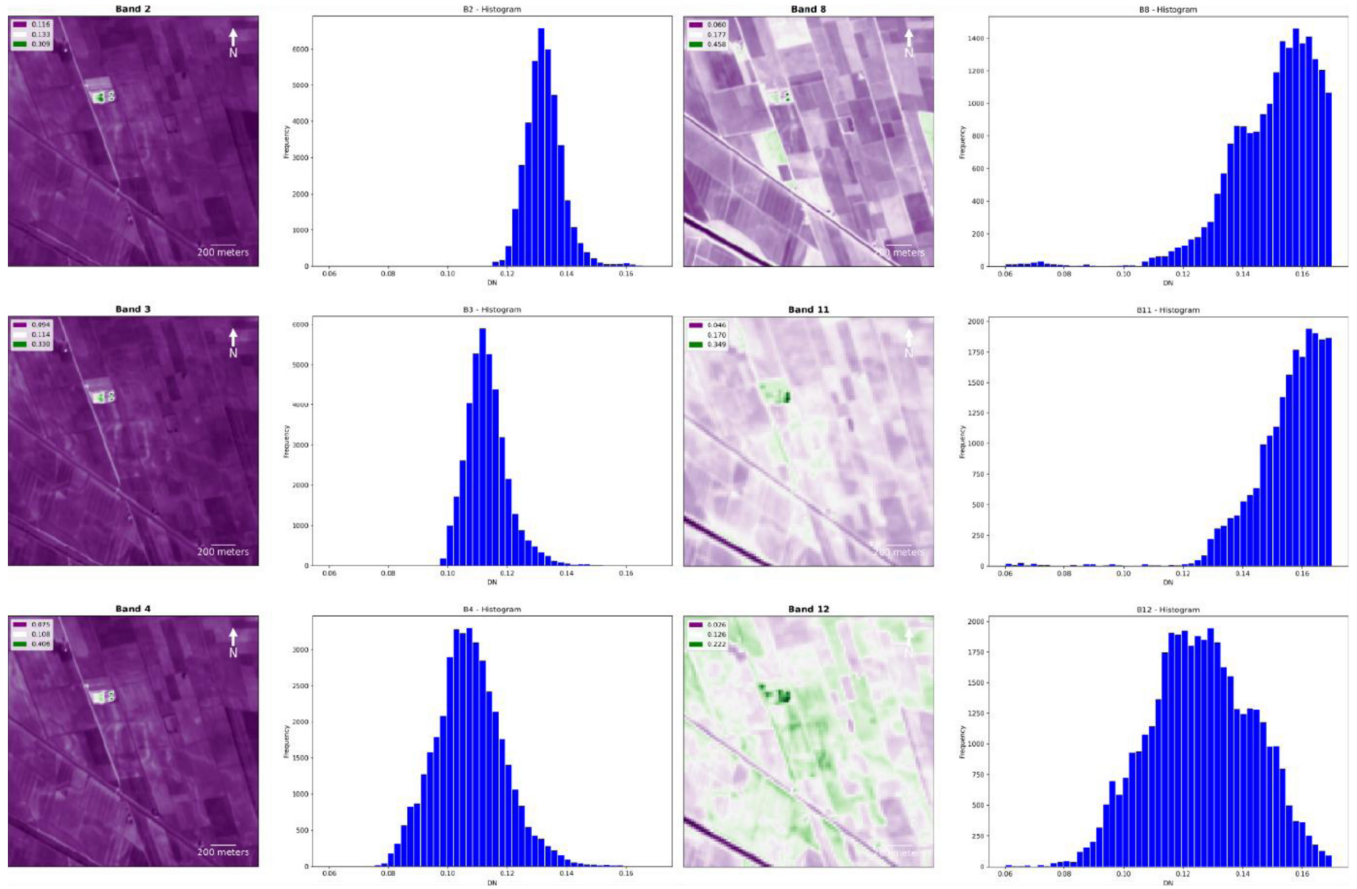
Fabbrica dei Soci site, plots and histograms of the bands B2, B3, B4, B8, B11 and B12.

The second part of the protocol enables the user to obtain plots and histograms for each output band. It is worth highlighting the usefulness of plotting each band of all outputs separately to compare the performance of single bands in the identification of crop/soil marks. In the example of Fabbrica dei Soci, Figure 13– Figure 17 show how some bands seem to offer a greater contrast between the palaeo features and the neighbouring spaces than the compositions/combinations, aiding the identification of buried elements. However, the visibility of these features is always dependent on several elements, and this may vary in other case studies. Moreover, histograms show the frequency distribution of the digital number (DN) values of each band, enabling a preliminary overview about the general performance of each output.

Looking at the singular band plot for the Fabbrica dei Soci site ( Figure 13), crop/soil marks seem particularly evident in the B4 and in the B8 plot even if significant differences in the B11 (SWIR 1) and B12 (SWIR 2) values have also been shown ( Figure 12). This is probably due to the higher resolution (10 m) of the B4 and B8 bands compared to the SWIR bands (20 m) ( Table 1).

**Figure 14.**
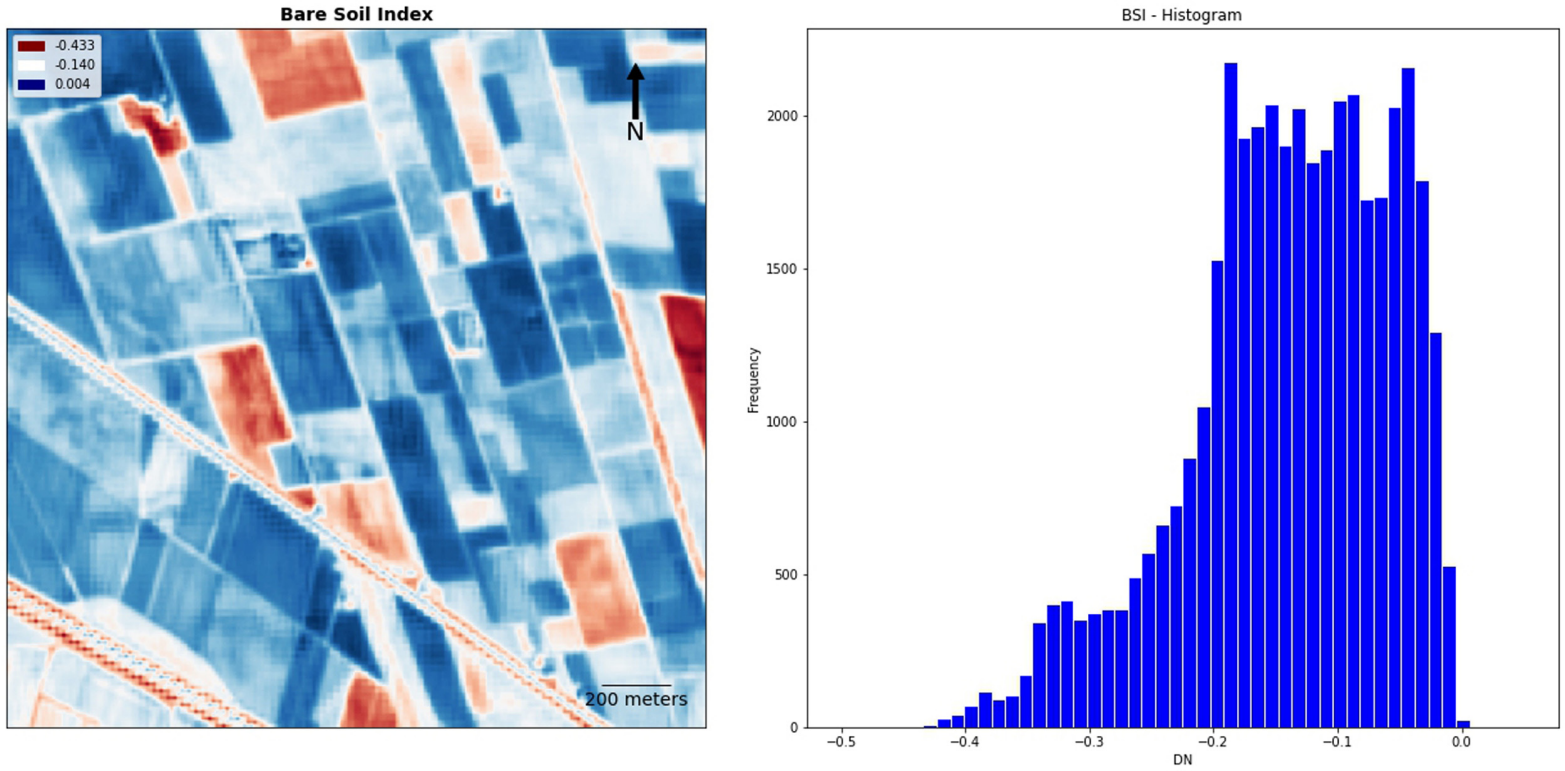
Fabbrica dei Soci site, plot and histogram of the Bare Soil Index (BSI).

The BSI index plot and histogram ( Figure 14) return no significant information about the overall performance of this output in the identification of related crop/soil marks: as mentioned above, the choice of this spectral index was not particularly fruitful to visualise buried palaeo-features except for TC buried structures such as moats and the village perimeter ( Figure 6 and Figure 9). 

**Figure 15.**
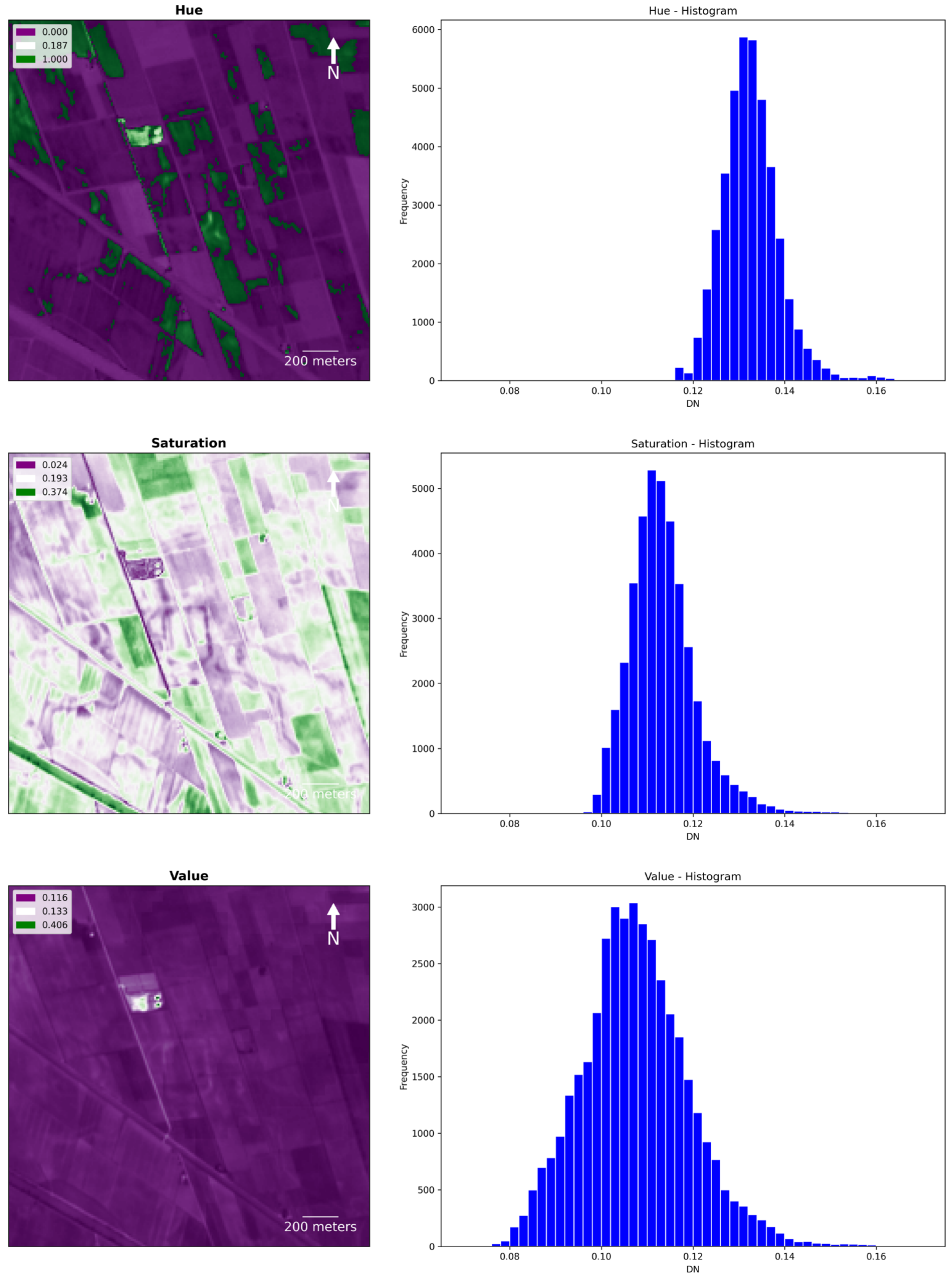
Fabbrica dei Soci site, Hue, Saturation and Value (HSV): plots and histograms of the bands Hue, Saturation and Value.

In, the HSV output the *Saturation* band returned the clearest visualisation of buried features ( Figure 15). As noted above the HSV consists of an alternative representation of the RGB colour space and vivid colours tend to be highly saturated while low saturation characterises pale colours.

**Figure 16.**
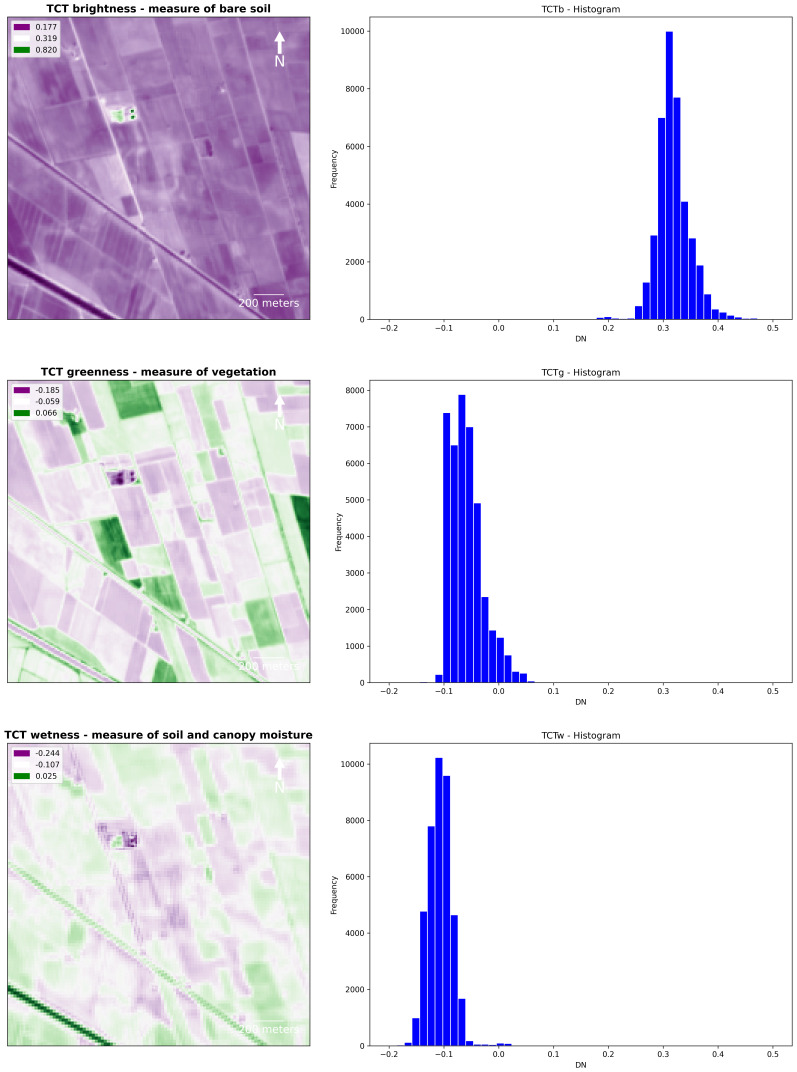
Fabbrica dei Soci site, Tasselled Cap Transformation (TCT): plots and histograms of the bands Brightness, Greenness and Wetness.

TCT and PCA were suitable for the identification of riverscape palaeo features in RGB combination. TCT was derived by the composition of TCTb, TCTg and TCTw bands and it was effective in the identification of positive crop/soil marks. In the *Brightness* band (TCTb, measure of bare soil), buried features are particularly visible, while they are barely recognizable in the *Greenness* (TCTg, measure of vegetation) and *Wetness* (TCTw, measure of soil and canopy moisture) bands ( Figure 16).

**Figure 17.**
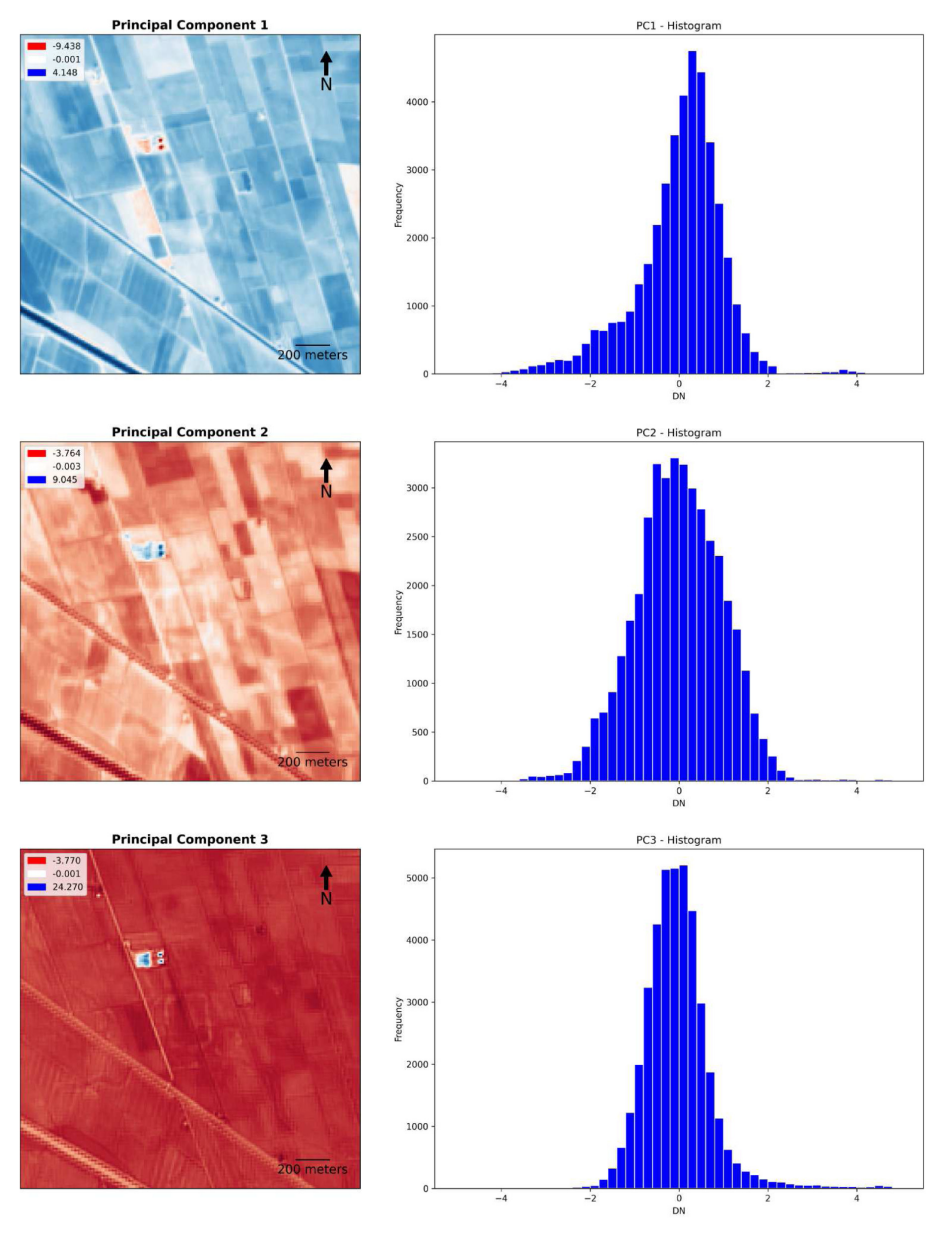
Fabbrica dei Soci site, Principal Component Analysis: plots and histograms of PCA 1, 2 and 3.

The detection of the palaeohydrography was much evident in the PCA obtained by the combination of the 1st, 2nd and 3rd principal components. PCA appears to be the most promising method adopted in this research along with the RGB and FSWIR SI composition. PCA outputs returned a detailed image of the riverscape palaeo-features in all the key points, considering that the first two or three principal components encompass nearly 80%–90% of the original data’s variance ^138^. Thanks to their capacity of reducing redundant information and highlighting variance for the recognition of individual elements, if we plot the PCA’s bands separately, some principal components depict a significant contrast between the background and the palaeochannels and buried canals which, in turn, substantially eases the detection of these features ^139^ ( Figure 17).

Considering the possible reproducibility of the method presented here, the main advantages of this FOSS-cloud protocol are not only limited to mitigating the need for specialist software and data licensing (thereby enabling a broader participation in the use of geospatial tools). One of the additional positive technical aspects of using the GEE Python API in Colab relates to computational power ( Table 3). For instance, both TCT and PCA are commonly considered time consuming methods especially when it is necessary to calculate large amounts of data. The Python script code tested in this research required less than a minute to calculate all the SI and SD outputs for each case study and the process could be run from any device regardless of the local machine specifications. That is possible because Colab is a hosted Jupyter notebook service that requires no setup to use, while providing free access to computing resources. The synergy between GEE, Python and Colab is extremely effective and versatile: essentially it is only necessary to change the region of interest (ROI) in the code script to calculate the SI/SD outputs in any area of the world ( Table 3). With the access to the GEE freeware planetary-scale satellite imagery dataset, our Python protocol could potentially be employed worldwide. Further, very basic coding skills are required to adapt the code to a ROI with different environmental characteristics or to customise the protocol with other SI formulas. In order to optimise the results it is only necessary to adapt the filtered image collection parameters to the peculiar environmental characteristics of the new ROI. Furthermore, the geemap Python module enables the interactive visualisation of the outputs directly in Colab: the images could also be stored and shared in Drive storage or downloaded to the local device for further analysis with GIS or graphical software. Finally, the modules rasterio, rioxarray and matplotlib enable the user to obtain downloadable plots and histograms for each output.

**Table 3.** Summary of the protocol advantages and limits discussed.

Advantages	Limits
Free and Open Access Datasets	Bands Resolution (max 10 m / pixel)
Freeware Cloud Computing	Preliminary analysis of environmental conditions of the study area is needed
Easy customisation (Basic skill coding required)	
Planetary coverage	
Interactive Spectral Signature Analysis	
Automatic extraction of frequency distribution of the digital number (DN) values of each output	

Nevertheless, besides the methodological advantages discussed, the current FOSS-cloud protocol still presents a few technical limitations ( Table 3). First, it is necessary to keep in mind that palaeo-landscape features smaller than 10 m (the maximum S2 band resolution) are hardly recognisable. Other remote sensing techniques such as airborne/terrestrial laser scanning and ground-based geophysical survey are considerably more efficient in revealing buried features ^17^ but these methods entail the use of equipment that is not always available, especially in remote areas of the world. Conversely, our FOSS - cloud protocol has a planetary coverage and the resolution limit could soon be overcome with the implementation of higher resolution datasets in the GEE collections or upsampling all the S2 bands with open-source tools such as Dsen2 ^139^. Secondly, the protocol does not automatically assess the best multitemporal period for analysis in a given area. In the test area, the choice of the timespan was based on previous scientific literature because the main research goal consisted only in verifying the potential of the FOSS-cloud protocol for the visualisation of known buried features. Nevertheless, any preliminary evaluations on a given study area will help to assess in which environmental conditions buried features are more likely to be detectable.

## Conclusion

Free and open source datasets of satellite imagery and freeware cloud computing offer considerable opportunities for landscape heritage stakeholders both for identifying features and monitoring changes. In this paper, a complete cloud procedure was developed as an alternative and versatile remote sensing FOSS method for the detection of palaeo-landscape features. S2 satellite imagery has been retrieved in the GEE dataset collection and analysed through a Python script code realized in Colab. Furthermore, the same script code enables the SI and SD analysis of the image collection, previously filtered to optimize the visualisation of crop/soil marks in different case studies in the Po Plain. The outputs obtained can be visualized directly in the Colab browser or downloaded via Google Drive for further graphical applications or spatial analysis.

Choosing the right timespan for a multitemporal analysis is crucial and it depends on peculiar environmental characteristics of the ROI. In the test area of the Po Plain, the chosen period was shown to be promising for the detection of crop/soil marks. The date range was based on information from previous literature and knowledge of local environmental characteristics: the protocol performance may be different in other studies and preliminary consideration of the environmental conditions of any ROI are required.

The highest discrimination capability was observed in RGB, FSWIR and PCA outputs enabling the recording of buried riverscape features. Most of these have been checked through the available geomorphological and archaeological literature; published case studies interpreting the occurrence of buried features served as a benchmark to validate visually the script code outputs. Finally, spectral signature values show the higher performance of the SWIR bands (B11 and B12) than the other bands considered in the identification of palaeo-features: this is likely to be due to their high sensitivity to moisture content and variations over time.

To summarise, the main advantages of this method consist of: i) being FOSS, all the software used here are open-licensed; ii) working in the cloud, no powerful hardware is necessary to run the script code; iii) high adaptability, changing the ROI is possible to calculate SI and SD outputs for any area of the world; iv) very basic coding skills are required to adapt the code to a ROI with different environmental characteristics. On the other hand, the current S2 image resolution represents a limit for the identification of archaeological structures smaller than 10 m. In addition, the protocol seems particularly effective in riverscape studies while its application in different environmental conditions is still unexplored. Nevertheless, the protocol can be customised with any of the spectral index formula available enabling a wide range of potential applications. Whilst some limitations persist ( Table 3), this FOSS-cloud protocol represents a potential alternative to remote sensing technologies such as lidar or geophysical survey which may be less accessible owing to technical or financial constraints. The development of FOSS-cloud procedures such as those described in this paper could support the identification, conservation and management of cultural and natural heritage anywhere around the world. In remote areas or where local heritage is threatened as a result of political instability, climate change or other factors, FOSS-cloud protocols could facilitate access to new data relating to landscape archaeology and heritage.

## Data availability

### Underlying data

*Google Earth Engine, Sentinel-2 MSI: MultiSpectral Instrument, Level-2A*

*https://developers.google.com/earth-engine/datasets/catalog/COPERNICUS_S2_SR*

### Terms of Use

*The use of Sentinel data is governed by the Copernicus Sentinel Data Terms and Conditions.*

### Extended data

Zenodo: *A Colab-Python script code to identify palaeo-landscape features*

https://doi.org/10.5281/zenodo.4384104 ^140^

This project contains the following extended data:

- GEEPY_PalaeoLandscape (Script allowing spectral indices and spectral decomposition analysis on Google Earth Engine satellite image collections).

Data are available under the terms of the Creative Commons Attribution 4.0 International Public License (CC BY 4.0).
