# Supplementary material for: A Google Earth Engine-enabled Python approach for the identification of anthropogenic palaeo-landscape features
Source: Open Res Eur. 2021 Sep 3;1:22. Originally published 2021 Mar 24. [Version 2] doi: 10.12688/openreseurope.13135.2 (PMC10445932; doi:10.12688/openreseurope.13135.2)
Supplement: Peer review history

# Peer review: Reviewer response for version 2

**DOI:** 10.21956/openreseurope.15177.r27528

**Published:** 2021-09-24

**Authors:** Tobias Ullmann

**Affiliations:** 1 Institute of Geography and Geology, Julius-Maximilians-Universitaet Würzburg, Würzburg, Germany

Dear authors,

 Thanks for the comprehensive revision of the manuscript. You addressed all the points I raised in my last report in a very good and reasonable manner. In my opinion, the quality of the manuscript has increased considerably and your contribution is now ready for publication.

 I have very few notes on the revised version (Version 2), which I suppose can be addressed during the final production. If not, these notes will be at least documented in this report:

 (1) “ ***Hue, saturation and value (HSV)***. *HSV (hue, saturation, value, also known as HSB or hue, saturation, brightness) is an alternative representation of the RGB colour space. In HSV [...]*“ --> You used B2, B3, and B4 for the HSV transformation, I suppose? For the sake of clearness that could be mentioned here, as the HSV color space can be used for any feature combination (not only for RGB).

 (2) ***"Principal component analysis (PCA)***. *The PCA transform (also known as the Karhunen-Loeve transform) consists of a linear transformation which decorrelates multivariate data by rotating the axes of the original feature space and outputs uncorrelated data ^120^. PCA [...]*“ --> Thanks for you comments and edits on this. The PCA results look most promising, in my opinion, and I welcome your discussion on the PCA and the assessments. However, here in the text it could be mentioned that the PCA was processed from all six bands (as you indicated in your answer, i.e. B2, B3, B4, B8, B11 and B12). Providing this information would avoid potential confusion, as your table 1 list all S2 bands. As well here you can mentioned how the PCA color composites (I mean the ones shown in the results, e.g. Figure 6) are composed. I suppose you used R=PC1, G=PC2, B=PC3?

 (3) **Figures in the results (Fig. 6 - 11):** If available, you can add information on the used stretch in the figure caption and the band combination for the TC and PCA color composites.

 Congratulations and thanks for the interesting work.

 Stay well and kind regards;

 Tobias Ullmann

If any, are all the source data and materials underlying the results available?

Partly

Is the work original in terms of material and argument?

Yes

Is the work clearly and cogently presented?

Partly

Is the argument persuasive and supported by evidence?

Partly

Does the research article contribute to the cultural, historical, social understanding of the field?

Partly

Does it sufficiently engage with relevant methodologies and secondary literature on the topic?

Yes

Reviewer Expertise:

Geomorphology, remote sensing

I confirm that I have read this submission and believe that I have an appropriate level of expertise to confirm that it is of an acceptable scientific standard.

# Peer review: Reviewer response for version 2

**DOI:** 10.21956/openreseurope.15177.r27527

**Published:** 2021-09-16

**Authors:** Richard Boothroyd

**Affiliations:** 1 School of Geographical and Earth Sciences, University of Glasgow, Glasgow, UK

Thank you for comprehensively addressing the comments raised in the initial review – I have no further comments.  The clarity and presentation of the revised article has been significantly improved.  I hope that the FOSS-cloud method will be widely used for detecting palaeo-landscape features.

If any, are all the source data and materials underlying the results available?

Yes

Is the work original in terms of material and argument?

Yes

Is the work clearly and cogently presented?

Partly

Is the argument persuasive and supported by evidence?

Yes

Does the research article contribute to the cultural, historical, social understanding of the field?

Yes

Does it sufficiently engage with relevant methodologies and secondary literature on the topic?

Yes

Reviewer Expertise:

Fluvial geomorphology and remote sensing.

I confirm that I have read this submission and believe that I have an appropriate level of expertise to confirm that it is of an acceptable scientific standard.

# Peer review: Reviewer response for version 1

**DOI:** 10.21956/openreseurope.14205.r26825

**Published:** 2021-05-21

**Authors:** Tobias Ullmann

**Affiliations:** 1 Institute of Geography and Geology, Julius-Maximilians-Universitaet Würzburg, Würzburg, Germany

The paper presents a python-based Google Engine approach that enables the processing of Sentinel-2 data for user-defined regions of interest. The script enables the processing of SI and SD. While the paper presents results for the detection of “anthropogenic palaeo-landscape features” in the Po plain, the script itself is not limited to be used for a specific application. The general topic of the work is of interest and the overall approach is sound and follows a clear logic. However, there are some issues that need be to addressed. There is a very positive and optimistic view on the presented approach, while at the same time it seems clear that there are some obvious limitations and drawbacks. The discussion should be extended and revisit the own results more critically.

- Introduction: It would be more stringent to present the objectives of the contribution at the end of the introduction.
- Figure 2: Provide full name of the abbreviations (BSI, HSV, etc.).
- Spectral indices, paragraph starting with *“Spectral indices that combine NIR and red channels generally…”*: Check guidelines on how to format equations. Not sure if these should stay in the text.
- Table 2: Use the band abbreviations (B1, B2…) you defined in Table 1?
- Sentinel-2 dataset; I was wondering about the cloud-masking, from my experience with the GEE and S-2 datasets there are problems to correctly mask out the clouds. Were similar findings made and is this an issue/limitation that should be reported in the discussion?
- Principal component analysis (PCA), *“Only 10-meter resolution bands were employed in PCA.”* - Why was the PCA constrained to these bands only? As mentioned above, the SWIR is of importance to highlight the crop/soil marks. So not including them might result in a loss of information?
- Figures 3 - 8: Consider placing one panel/subfigure that just shows the archaeological records. Right now, there is no chance for the reader to independently judge whether a feature is visible in the data or not. How can you be sure that the arrows indicate palaeochannels and canals? Or is this interpretation taken from the imagery? This is not clear form the figure.
- Discussion; *“this alternation of high and low rainfall intensity enabled the calculation of the mean values of multitemporal bands significant for the identification of soil marks.” *- This issue is not elaborated and not investigated. You have not checked what happens with the results when cancelling some years.
- Discussion; *“In all six case studies the best performance with respect to the SI outputs was provided by the RGB combination.” “Among the SD techniques tested in this study, the HSV outputs enabled the clearest identification of palaeochannels; as noted above the HSV consists of an alternative representation of the RGB colour space.*” - This is your expert judgement, there is no quantitative data/analysis that would support these statements.
- Discussion; *“PCA outputs returned a detailed and clear image of the riverscape palaeo-features, considering that the first two or three principal components encompass nearly 80 to 90% of the original data’s variance” *I wouldn’t call it a detailed and clear image, as still the identification of features relies purely on expert interpretation. Besides, as mentioned in the text the PCA was computed from the 10 m bands as such the statement on the 80-90% original data’s variance is misleading as SWIR etc. were not considered?
- Discussion; As finally the expert is identifying the features, I suggest avoiding statements like *“with more accuracy”, “decreasing the occurrence of false positives”* or *“significant”* as there are no quantitative analyses that would prove such findings.
- Figure 9: Colour bar/legend missing for the PCs. It is not possible to judge on the scaling from this representation. The bin size of the histograms looks to large, i.e. the true shape of the distribution is not visible. Consider redrawing the histograms and use a smaller bin size and a more fitting min/max.
- Discussion and Figure 10: *“the values of the bands that provide a better performance are those with the values more clustered, as depicted in the histograms (Figure 10)” -* It is not clear to me why the histograms should support this statement. Why should it be possible to judge on the performance in feature detection form the histograms? As the frequency of feature occurrence is much small than the frequency of the non-occurrence, some might expect the features of interest to be visible in some later PCs?
- Figure 10: Typo in *“Plots (Above) and histograms (below)”*, it is the other way round. Colour bar/legend missing.
- Discussion; How do authors see the transferability to other regions in the world, as this is mentioned in the conclusion. What problems and limitations will arise?
- Conclusion; *“methodology proposed is very effective in the reconstruction of Mid-Late Holocene landscape evolution of the Po Plain” -* this is a very strong statement that is not supported by your analysis. There is no landscape reconstruction, as well even though features can be detected by means of remote sensing, no information on the date/time etc. is available just from the imagery, but would be needed for reconstructing the landscape evolution.

If any, are all the source data and materials underlying the results available?

Partly

Is the work original in terms of material and argument?

Yes

Is the work clearly and cogently presented?

Partly

Is the argument persuasive and supported by evidence?

Partly

Does the research article contribute to the cultural, historical, social understanding of the field?

Partly

Does it sufficiently engage with relevant methodologies and secondary literature on the topic?

Yes

Reviewer Expertise:

Geomorphology, remote sensing

I confirm that I have read this submission and believe that I have an appropriate level of expertise to confirm that it is of an acceptable scientific standard, however I have significant reservations, as outlined above.

# Response

**Published:** 2021-08-23

**Authors:** FILIPPO BRANDOLINI

**Affiliations:** Newcastle University, UK, UK

We are grateful for your comments and suggestions that helped us to improve the paper considerably. In general, we tried to clarify the main aim of this paper: presenting an alternative FOSS - Cloud tool useful in detecting buried palaeolandscape features. In the first version, we did not explain clearly enough that the Po Plain served only as a test case area. Our intention was to use these well-known archaeological areas only to assess the general performance of the protocol visually. The images and the text have been improved to explain this research goal more effectively.

 Following your suggestion, we added an interpretative drawing indicating all the buried features known in the literature. This improved the quality of the images significantly. Also, we clarify in the text that the cloud masking has been performed using the default S2 QA60 band provided in GEE. In relation to the PCA, we agree that considering only the 10m bands was an error: we apologise for this inadvertent problem in the code (then reported in the text!). We fixed the PCA part of the script code, and now all six bands are employed in the analysis. In the Discussion, we added more data for a general overview of the performance of each output band. Indeed, we updated the script code that now returns plots and histograms of each band automatically. Even if this does not serve as a quantitative analysis to assess the performance of detecting buried features in the Po Plain specifically, it represents a useful tool for any users to have a preliminary overview of the general performance of the outputs.

 Also, the Inspector/Interactive Plot tools provided by the geemap Python module enable the spectral signature analysis to assess which bands tend to perform better in the identification of buried features. We did not want to focus on mapping new buried features in the test case area but to show the potential and limits of FOSS and cloud resources in doing that. Consequently, we think that our main research aim is much clearer than in the first version of the paper. Finally, following your suggestion, we avoided inappropriate statements in the Discussion and Conclusion, and we added more consideration about the general pros/cons of the FOSS - cloud method proposed.

# Peer review: Reviewer response for version 1

**DOI:** 10.21956/openreseurope.14205.r26622

**Published:** 2021-04-28

**Authors:** Stefano Campana

**Affiliations:** 1 Department of History and Cultural Heritage, University of Siena, Siena, Italy

The title does not fully match the topic of the paper. In particular it does not indicate that the automatic identification under discussion relies exclusively on the spectral signature of the images and not on other fundamental criteria usually implemented in the identification process, such as shape, size, association etc. In addition to the problem of the title, the original decision to base the identification of anomalies purely on the spectral characteristics is extremely questionable and barely acceptable as a research methodology.

 The first section ( *Toward a definition of “landscape heritage”*) seems to me very generic and for this reason inappropriate. I would rather suggest introducing straightforward the subject of the paper. The second section ( *GIS and remote sensing in landscape studies: the satellite 'revolution'*) contains questionable statements with which many researchers/scholars in this field would profoundly disagree. In particular, perhaps, “satellite imagery has dramatically improved the quality[?] of the historic landscape characterization (HLC) approach”. The authors need to explain in what sense it has done this, and in what spheres of operation? In terms of both quantity and quality of the information obtained how does it compare with what is available from other sources? For instance, in Europe but also in many other countries that have large archives of aerial photographs and/or alternative remote sensing data (such as lidar) that are accessible to specialists and the general public, and where aerial survey can be undertaken in the present day, satellite images continue to have a very limited usage in landscape studies. It is undeniable that for each site or context identified through satellite imagery vastly greater numbers have been identified (in the same areas) through aerial photography. In fact, the authors' claim is valid in only in those countries where aerial photographs and other airborne or remote-sensing datasets are not available, for instance in most of the countries in the MENA region, along with Turkey, China and Russia to name but a few.

 Furthermore, in the introduction, among the reasons "why riverscapes?" the authors do not mention an absolutely crucial consideration in any form of remote sensing – that of geometric resolution. There is indeed a close relationship between the dimensions and structural characteristics of paleo-riverbeds and the spatial resolution of the satellite imagery used by the authors. However, most meaningful archaeological traces, apart from the largest enclosed settlements or ritual sites, are too small to be identified in the kind of satellite imagery that are under discussion here. Paleo-riverbeds or paleochannels are by contrast among the most widespread and easily observable features that can be recorded by this kind satellite imagery – for instance in the Po Valley in northern Italy. For the sake of clarity this should be clearly acknowledged in the text.

 The reasons for focusing the research on soilmarks rather than cropmarks are unconvincing. In the section on "Material and methods" the authors state that "the detection of crop marks is affected by several issues, the most important is the phenological stage of the crops". Another influence, of course, is luck – the ‘serendipity’ of being in the right place to record the cropmarks when they are readily detectable – a problem which applies to any form of aerial or satellite recording which acquires imagery at a particular moment in time rather than (perhaps) at regular intervals throughout the whole course of the changing annual seasons. It is widely acknowledged that cropmark phenomena (in European landscapes) represent the most numerous source of recordable archaeological features, vastly greater than the total amount of evidence identified by any other form of remote sensing (apart, perhaps, from large-scale geophysical prospection in carefully chosen landscapes). Cropmarks provide by far the most easily identifiable archaeological traces for a very simple reason: when they are present they remain visible for a significant period of time, typically from two to six weeks in one degree of clarity or another. Moreover, throughout this time they remain visible at all times of day, from early morning to late evening. Furthermore, it is now being shown that multispectral imagery can also detect them significantly before they become visible to the naked eye (and to traditional monochrome or colour photography). Soilmarks, by contrast, are the most difficult category to identify because of their ephemeral appearance or disappearance in response to the often-transient balance between dry and wet soils under the impact of local weather conditions and the varying processes of arable cultivation. Considering the avowedly multitemporal perspective of the project, the reasons for the choice of the period appear highly questionable.

 The Figures, at their present scale, are barely compressible, so small and in such low contrast that it is barely possible to see the arrows and the supposed anomalies are barely visible at all. Only in Figure 7 do there appear to be anomalies which could with reasonable certainty be associated with paleochannels.

 In my view both the text and illustrations need considerable improvement before they would be worthy of indexing. A highly desirable addition would be a more realistic assessment of what can (or cannot) be achieved, in what kinds of contexts and geographical areas, by this approach to the analysis of satellite data.

If any, are all the source data and materials underlying the results available?

Partly

Is the work original in terms of material and argument?

Partly

Is the work clearly and cogently presented?

Partly

Is the argument persuasive and supported by evidence?

Partly

Does the research article contribute to the cultural, historical, social understanding of the field?

Partly

Does it sufficiently engage with relevant methodologies and secondary literature on the topic?

Partly

Reviewer Expertise:

Landscape archaeology and remote sensing in archaeology.

I confirm that I have read this submission and believe that I have an appropriate level of expertise to confirm that it is of an acceptable scientific standard, however I have significant reservations, as outlined above.

# Response

**Published:** 2021-08-23

**Authors:** FILIPPO BRANDOLINI

**Affiliations:** Newcastle University, UK, UK

Thank you for your helpful considerations of our paper. We agree that the research goal was not clearly explained in the first version and some parts of the manuscript needed to be improved. In the introduction, we explain why we decided to focus on FOSS and Cloud tools in landscape studies, supporting our considerations with the most recent literature on the argument. In this regard, we agree with you that it is not correct to talk about a “satellite revolution” in remote sensing studies. One of the greatest improvements that have been observed in recent years is the advent of FOSS-cloud platforms that provide both open access data and freeware computational power: we reported a few of the most recent publications about the successful application of these technologies in landscape archaeological studies.

 Furthermore, following your suggestion, we revised the manuscript section discussing the identification of crop/soil marks. In the first version, it was not clear that we focused on a low-vegetation period (basing that decision on previous scientific literature). Also, we mentioned only the soil marks in the first version of the paper even if some crop marks were evident in the images provided. We have rewritten the manuscript to clarify our intention. Moreover, we agree with you that soil marks are the most difficult category to identify because of their ephemeral appearance or disappearance in response to the often-transient balance between dry and wet soils under the impact of local weather conditions and the varying processes of arable cultivation. That is absolutely true in the case of aerial images. Still, one of the main advantages of a satellite multi-temporal approach is to calculate the mean pixel value of a given period. In this case, the choice of the multi-temporal is based on previous scientific literature in which low-vegetated periods of the year seem to enable better detection of buried features in the Po Plain. In addition, we added new charts (Material and Methods) in which the GEE satellite dataset shows the date range that corresponds to low vegetated periods in the Po Plain.

 Finally, we revised the Discussion and Conclusion along with illustrations to clarify that the paper's aim consists of proposing an alternative FOSS-cloud remote sensing tool rather than mapping new buried features in the Po Plain. The test case area served only to show the advantages and disadvantages of this remote sensing protocol for landscape studies. Comparing the spectral signatures of buried palaeo features and neighbouring area reveals significant differences in the SWIR bands’ values.  This result indirectly confirms that the multi-temporal period chosen is particularly effective in the identification of buried features since the SWIR spectral range is strongly sensitive to soil moisture content and variations over time.

# Peer review: Reviewer response for version 1

**DOI:** 10.21956/openreseurope.14205.r26697

**Published:** 2021-04-20

**Authors:** Richard Boothroyd

**Affiliations:** 1 School of Geographical and Earth Sciences, University of Glasgow, Glasgow, UK

The research article provides an interesting application of a Google Earth Engine-enabled Python approach for identifying palaeo-landscape features on the Po Plain, Italy. Using Sentinel-2 satellite imagery, the study presents a freely accessible and open-source methodology for detecting and interpreting buried features in the landscape. Several locations on the Po Plain with well-known archaeological sites are used to test the methodology, with excellent descriptions of the sites provided. In general, the methodology is clearly described and logical to follow. For each site, palaeo-landscape features (e.g., palaeochannels and canals) are detected and interpreted, with discussion of the advantages and disadvantages of the different identification approaches (including spectral indices and spectral decomposition). Overall, the study provides an important research contribution that is of interest to a range of audiences (both technical and general).

 The study is original in terms of material and argument. Although established spectral indices and spectral decomposition techniques are used, the multitemporal element for detecting palaeo-landscape features is original. The study engages with relevant methodologies and secondary literature on the topic (spanning heritage, remote sensing and fluvial geomorphology literature).

 Parts of the results section could be more clearly presented. Specific suggestions are made to improve the interpretation of figures (in particular adding colour bars and re-considering the choice of symbols to delineate buried features with palaeoflow directions). Colour bars would be needed for the Figures to be scientifically sound.

 On the whole, the argument is persuasive and supported by evidence. Formal accuracy assessments could be added to quantitatively assess the performance of the methodology. Sensitivity analysis could be undertaken to assess the effect of shifting the date range (i.e., from autumn-winter to spring-summer). These suggestions would strengthen some of the assertions made in the conclusions but could be deemed beyond the scope of the current ‘proof of concept’ work. Consideration for some of the methodological limitations could be added to the discussion – i.e., under what scenarios/environmental conditions does the methodology perform less well? This could help researchers to assess whether the methodology is suitable for their own study sites.

 Source data and materials underlying the results are available – the code is accessible, well documented and easy to run using Google Colab. The source code will be a useful resource for researchers working across multiple fields and can be easily modified to apply the methodology to different regions of interest. 

**Specific suggestions:**

- Introduction, paragraph 6 – “Archaeological investigations have confirmed that over the last 5000 years human activities have profoundly altered the spatial configuration and rate of fluvial and alluvial geomorphic processes”. From a fluvial geomorphology perspective, it is unusual to see the term ‘alluvial geomorphic processes’ – you could replace this with ‘fluvial processes’ (to indicate the processes of erosion and deposition).
- Study area, paragraph 2 – “Intense rainfall (700–1200 mm per year) occurs throughout the year and the seasonal pattern of precipitation strongly influences the annual regime of the Po River” – in addition to rainfall, it would be worthwhile to mention the snowmelt component important to the annual regime (e.g., Montanari, 2012) ^1^.
- Materials and methods, paragraph 1 – “The mosaic of cultivated ﬁelds on the Po Plain changes all the time which makes uniform visual analysis difficult”. The meaning is a little unclear here, does this refer to only the visible spectrum (i.e., RGB)? Are the same challenges experienced when using multispectral data?
- Materials and methods, paragraph 5 – “To help overcome this issue, this study adopted a multitemporal approach by calculating the mean values of bands over two ninety-day periods (January–March and October–December) of each year from 2015–2020.” I was confused about the outputs here – do you produce a single image for the entire analysis period, or several annual composite images? Could you add an extra summary sentence to help the reader understand the output, e.g., ‘The workflow generates a single composite image for the entire analysis period (2015-2020) containing x bands.’
- Materials and methods, paragraph 5 – “(January–March and October–December)”. Was any sensitivity testing undertaken to assess the effect of changing the date range? This could be beyond the scope of the current paper, but what effect does shifting the date range +/- 1 month or +/- 3 months have on the detection and interpretation of buried features? Sensitivity testing could provide more robust evidence to support the claims made in the conclusion.
- Materials and methods, paragraph 5 – the cloud masking procedure is not reported in the methods section but is an important step in the workflow. It would be useful to add a sentence indicating how cloud and cloud-shadow pixels were masked.
- Materials and methods, Principal component analysis (PCA) section – for TCT, you specify each band of the 6-Band Image. For completeness in the PCA section, could you specify the bands included in the 4-Band Image (R, G, B, Nir).
- Figures 3-8 – What is the rationale for not including colour bars in the figures? Adding colour bars could aid interpretation (e.g., for BSI, it is unclear whether the buried structures are indicated by locally high or locally low values). Related to this, are the values used to limit the colour maps the same between the figures (i.e., are the minimum-maximum values for BSI the same throughout Figures 3-8)?
- Figures 3-8 – Do the arrows indicating palaeochannels and canals align with the palaeoflow directions? If not, an alternative symbol (e.g., x or *) might better delineate these features so as not to imply a palaeoflow direction.
- Figure 9 – Would a colour bar be helpful here? Could more descriptive subplot titles help guide the reader (currently PC1, PC2, PC3, etc). Alternatively, could this description be included to the figure caption?
- Figure 10 – Would a colour bar be helpful here? Could more descriptive subplot titles help guide the reader (currently B2, B3, B4). Alternatively, could this description be included to the figure caption?
- Discussion, paragraph 5 – “Just like the RGB combination, whose B3 – green and B4 – red bands depict the palaeoenvironmental features with more accuracy”. Without including a formal accuracy assessment, I think it is risky to comment on the ‘accuracy’ of the feature detection (i.e., what about those features that are undetected by the methodology?). Rather than referring to accuracy, it could be helpful to discuss in terms of aiding the interpretation.
- Conclusion, paragraph 2 – “The choice of the autumn-winter period was shown to be effective for the detection of soil marks in the Po Plain. Choosing the right timespan for a multitemporal analysis is crucial”. This is an important point, but not fully supported because other time periods have not been presented/discussed (i.e., are fewer features detected if a spring-summer time period is used?). This could be rephrased to reiterate the importance of considering environmental conditions (e.g., drought) when selecting the time period.
- Conclusion, paragraph 3 – “In general, the methodology proposed is very effective in the reconstruction of Mid-Late Holocene landscape evolution of the Po Plain.” This sentence overstretches the findings of the study (reconstruction and landscape evolution infers some knowledge of the sequence of events – I don’t see how the results support the statement). It could be rephrased to something more general on the utility of the tool, e.g., ‘In general, the proposed methodology is a useful tool to detect and interpret palaeoenvironmental features in the fluvial landscape of the Po Plain’.
- Conclusion, paragraph 3 – “ii) working in cloud”. Missing word ‘the’.

If any, are all the source data and materials underlying the results available?

Yes

Is the work original in terms of material and argument?

Yes

Is the work clearly and cogently presented?

Partly

Is the argument persuasive and supported by evidence?

Yes

Does the research article contribute to the cultural, historical, social understanding of the field?

Yes

Does it sufficiently engage with relevant methodologies and secondary literature on the topic?

Yes

Reviewer Expertise:

Fluvial geomorphology and remote sensing.

I confirm that I have read this submission and believe that I have an appropriate level of expertise to confirm that it is of an acceptable scientific standard, however I have significant reservations, as outlined above.

## References

[1] : Hydrology of the Po River: looking for changing patterns in river discharge. *Hydrology and Earth System Sciences* .2012;16(10) : 10.5194/hess-16-3739-2012 3739-3747 10.5194/hess-16-3739-2012

# Response

**Published:** 2021-08-23

**Authors:** FILIPPO BRANDOLINI

**Affiliations:** Newcastle University, UK, UK

Thank you for your constructive criticism. Following your comments and suggestions, the general quality of the paper was enhanced considerably. Firstly, following your suggestion, we improved the general quality of the figures. In the Discussion, we added colour bars as you suggested for each band plot and the corresponding histogram. Furthermore, we added a spectral signature analysis to assess which bands tend to highlight the palaeolandscape features better.

 Regarding the symbology used to delineate buried features, we preferred to add an interpretative drawing with all the known buried features along with each site’s outputs. We think that in this way, the buried features can be recognised more easily; we chose this solution because adding symbols directly to the outputs could make the visualisation difficult for readers. Moving all the symbols into a separate interpretative drawing avoids this problem. Also, we clarify in the text that the cloud masking has been performed using the S2 QA60 band.

 We agree that a sensitivity analysis would be necessary to assess the effect of shifting the date range but consider this to be beyond the scope of this paper. In the first submitted version it was perhaps not explained clearly enough that the main goal was to present an alternative FOSS-cloud procedure to detect buried features. The Po Plain served only as a test case area to show the protocol's performance, so the choice of the archaeological sites and the timespan was based on scientific literature. We have tried to clarify our intentions in this respect in the revised paper. We have also added new bibliographic references in which authors indicated low - vegetation periods as better conditions to identify buried features in the test case area. Also, three new figures have been added in the Material and Methods section to support our decision to consider the low vegetation period as ideal for the test case area.

 Consideration about the phenological stage of crops and field mosaicing are two common issues documented in similar published studies which we refer to in the manuscript. We have updated the script code that now returns plots and histograms of each band automatically. Even if this does not serve as a quantitative analysis to assess the performance of detecting buried features in the Po Plain specifically, it represents a useful tool for users, providing a preliminary overview of the general performance of the outputs. We did not want to focus on mapping new buried features in the test case area but to show the potential of FOSS and cloud resources to do so. In this regard, following your suggestion, we have added some considerations about methodological limitations. The two main limits are the low resolution of outputs, especially in identifying features smaller than 10 m, and the choice of the period: the latter depends on the environmental conditions of each study area, but it is something that our FOSS-cloud protocol still cannot assess automatically. To conclude, we hope that the paper's aims and conclusions are better explained and supported by the literature referenced, the images and the tables.
